# Prognostic value of NT-proANP levels on major cardiovascular outcomes in a 31-year follow-up study depends on baseline morbidity

**DOI:** 10.1038/s41598-025-03819-6

**Published:** 2025-05-28

**Authors:** Samuli Sakko, Juha Perkiömäki, Antti Ylitalo, Heikki Huikuri, Olavi Ukkola, Peppi Koivunen, Joona Tapio

**Affiliations:** 1 Biocenter Oulu and Faculty of Biochemistry and Molecular Medicine, Research Unit for Extracellular Matrix and Hypoxia, University of Oulu, P.O. Box 5400, 90014 Oulu, Finland; 2 Research Unit of Biomedicine and Internal Medicine, Medical Research Center Oulu, University of Oulu and Oulu University Hospital, Oulu, Finland; 3 Turku University Hospital, Heart Center, Turku, Finland; 4 University of Turku, Turku, Finland

**Keywords:** NT-proANP, Cardiometabolism, Cardiovascular disease, Insulin resistance, Heart failure, Cardiology, Diseases, Medical research

## Abstract

In subjects with cardiovascular disease (CVD), higher N-terminal proatrial natriuretic peptide (NT-proANP) are associated with major cardiovascular outcomes, while in subjects without CVDs longitudinal data is largely lacking. Our aim was to assess cross-sectional associations between NT-proANP and key CVD factors (1044 subjects, 40–62 years, 51% hypertensive, 49% males) and to evaluate the predictive potential of NT-proANP for HF events, CVD events, CVD mortality, and total mortality over an up-to 31-year follow-up period. In subjects with CVDs, the high NT-proANP tertile had decreased kidney function, higher prevalence of CVDs and adverse echocardiographic measures but also the lowest fasting insulin levels, and in longitudinal analysis had an increased risk for HF events and CVD mortality. In subjects without CVDs the high NT-proANP tertile had the healthiest metabolic profile with the lowest BMI, fasting insulin levels and blood pressure, and in longitudinal analysis weaker evidence for increased risk for HF events and CVD mortality were observed. Regardless of CVDs, NT-proANP levels increased with age and were not independently associated with total mortality. In this middle-aged population, followed for up to three decades, the cross-sectional and longitudinal associations of NT-proANP levels were largely dependent on population characteristics such as age and CVDs.

**Supplementary Information:**

The online version contains supplementary material available at 10.1038/s41598-025-03819-6.

## Introduction

Atrial natriuretic peptide (ANP) is a peptide hormone secreted from the cardiac atria in response to increased atrial wall strain, aiming to restore fluid balance^1^. The N-terminal proatrial natriuretic peptide (NT-proANP), co-secreted with ANP, is inactive but more stable having half-life of 60 to 120 min compared to 5 min of ANP^2^, thus more reliably measured from the circulation^1^. Plasma NPs, especially B-type (BNP) and N-terminal proBNP (NT-proBNP), are widely utilized as a prognostic and diagnostic tool in heart failure (HF)^3,4^. Based on clinical findings reported at the turn of the century^5–7^, BNP and NT-proBNP have become the more widely used measure of the NPs.

Like other NPs, in cohorts with pre-existing CVDs, higher levels of NT-proANP have shown predictive value for a variety of outcomes including HF^6,8–10^, atrial fibrillation (AF)^8,11^, chronic heart disease (CHD) events^8–10^, and mortality^8–10,12,13^. However, in contrast with these longitudinal data, in the Framingham Offspring study, in subjects without CVDs (not including hypertension or diabetes), higher levels of NT-proANP were cross-sectionally associated with an ideal cardiovascular health score^14^, suggesting the prognostic value of NT-proANP might be dependent on study population characteristics. Additionally, lower levels of NT-proANP have been cross-sectionally associated with various metabolic risk factors including unhealthier body composition^15–18^, hyperinsulinemia^16–18^ and longitudinally with development of diabetes (DM)^19^. To further support these findings, similar observations have been reported for MR-proANP^20,21^ and NT-proBNP^20^ with potential causal evidence^22,23^. As these metabolic factors are key risk factors of CVDs, the utility of NT-proANP in the prognosis of CVD-related outcomes, especially in subjects without established CVDs, requires further research.

Utilizing a cohort of 1045 Finnish subjects (age 40–62 years at baseline) followed for up to 31 years, this study aimed to (1) assess cross-sectional associations between NT-proANP and key CVD factors, including echocardiography and liver sonography, in middle age (average age 51.3 years), and (2) longitudinally evaluate the predictive potential of NT-proANP measured in the middle-age for HF events, cardiovascular (CVD) events, CVD mortality, and total mortality over an up-to 31-year follow-up period in whole population analysis and in subjects without and with CVDs.

## Materials and methods

### Study population

A flow-chart of the study is presented in Fig. S1. Oulu Project Elucidating Risk of Atherosclerosis (OPERA) is an epidemiological cohort study addressing CVD risk factors and clinical endpoints. Subjects (*n* = 1045, 50% hypertensives, 50% males, age 40–62 years), were randomly selected between 1990 and 1993 as middle-aged hypertensives, and their age- and sex-matched controls, as previously described in detail^24^. At baseline, subjects were interviewed, examined and tested in the University of Oulu research laboratory. Mortality and hospital events of the study population have since been followed.

Inclusion criteria for the current study were available NT-proANP levels (*n* = 1044) at baseline. For a subpopulation analysis, the population was divided based on morbidity and medications at baseline. The subpopulation without CVDs, DM, chronic kidney disease (CKD) or related medications included a total of 410 subjects. The subpopulation with CVDs, DM, CKD or related medications included a total of 634 subjects. Of the 1044 subjects, 631 were alive by the end of 2021 (current analysis period), with 139 of 413 deaths classified as CVD-related.

The study adheres to the Declaration of Helsinki and was approved by the Ethics Committee of the Faculty of Medicine, University of Oulu. Written informed consent was obtained from each participant.

### Background information

Lifetime smoking burden was determined in pack-years (1 pack-year = 20 cigarettes smoked/day for 1 year) from a questionnaire. Alcohol consumption was reported as standard drinks/week and converted to g/wk. Medications were determined from patient records. Hypertension (HTN) was diagnosed based on the national registry of the reimbursement of the costs for antihypertensive medication^25^ or blood pressure over 140/90 at baseline. DM was defined as a known diagnosis (fasting plasma glucose of ≥ 7.0 mmol/l or 2 h plasma glucose of ≥ 11.1 mmol/l in the oral glucose tolerance test (OGTT)) at baseline^26^. Coronary artery disease (CAD) was based on patient records of prior CAD events and electrocardiogram (ECG) criteria at baseline. Diagnosis CKD was based on patient records of prior diagnosis or estimated glomerular filtration rate (eGFR) < 60 ml/min/1.73 m^2^ at baseline. Diagnoses of prior acute myocardial infarction (AMI), stroke and transient ischemic attacks (TIA) were based on patient records.

### Clinical examination

All measurements were performed by specially trained nurses. Body mass index (BMI) was calculated as weight (kg) divided by height squared (m^2^). Height was measured to the nearest centimeter (cm) without shoes using a stadiometer, and weight was measured to the nearest 0.1 kg with light underwear without shoes using a calibrated SECA personal scale.

### Laboratory analyses

Most laboratory analyses have been earlier described in detail^27^. Blood samples were analyzed at NordLab Oulu (former name Oulu University Hospital, Laboratory), a testing laboratory (T113) accredited by Finnish Accreditation Service (FINAS) (EN ISO 15189).

NT-proANP levels were assayed directly from plasma. Standards of synthetic human proANP-(79–98) and samples (25 μl) in duplicate were incubated with 200 ul of radioiodinated human Tyr-(O)-proANP-(79–98) and 200ul of rabbit antiserum 135 (final dilution l/40,000) overnight at + 4”C. The bound and free fractions were separated by double antibody precipitation in the presence of polyethylene glycol. The sensitivity of the assay was 40 pmol/L plasma and the within and between assay coefficients of variation were < 10% and < 15%, respectively. Gel filtration high-performance liquid chromotographic analyses of plasma samples consistently showed the presence of only one immunoreactive species corresponding in size to that of intact NT-proANP (NT-proANP 1-98)^28^.

Blood leucocytes were measured using fluorescence flow cytometry, and blood hemoglobin (Hb) levels using the sodium lauryl sulfate method. eGFR was estimated using formulas described by Inker et al.^29^ Glucose concentrations were measured using glucose dehydrogenase method (Diagnostica, Merck, Darmstadt, Germany) and serum insulin levels with a two-site immunoenzymometric assay (AIA-PACK IRI, Tosoh Corp., Tokyo, Japan). Homeostatic model assessment for insulin resistance (HOMA-IR) was calculated with the equation (fasting insulin (mU/mL) × fasting glucose (mmol/L)/22.5).

Very-low-density lipoprotein (VLDL) fraction was separated from plasma by ultracentrifugation at 10,500 × g for 18 h. Plasma high-density lipoprotein (HDL) cholesterol concentration was measured by mixing 0.5 mL of the VLDL-free fraction with 25 mL of 2.8% (wt/vol) heparin and 25 mL of 2 M manganese chloride and measuring the cholesterol concentration in the supernatant after centrifugation at 1000 × g and 4 °C for 30 min. Low-density lipoprotein (LDL) cholesterol concentration was calculated by subtracting the HDL cholesterol concentration from the VLDL-free fraction.

### Ambulatory blood pressure

Ambulatory blood pressure (ABP) was measured with a non-invasive fully automatic SpaceLabs90207 oscillometric unit (SpaceLabs Inc., Redmond, WA) every 15 min from 04:00 a.m. to 12:00 p.m. and every 20 min from 12:00 p.m. to 04:00 a.m. The accuracy of bp readings obtained have previously been settled by the British Hypertension Society and the US Association for the Advancement of Medical Instrumentation^30^. The siemilarity (difference < 5 mmHg) between four BP measurements and four auscultatory readings using a Y-connector was required to ensure proper positioning of the cuff. Study subjects were instructed to relax their arm during the measurement. Systolic BP values ≤ 70 mmHg or ≥ 250 mmHg, diastolic BP values ≤ 40 mmHg or ≥ 150 mmHg, and heart rate ≤ 40 or ≥ 150 beats/min were automatically excluded. Based on these criteria, less than 3% of measurements were excluded^31^.

### Echocardiography

The echocardiographic measurements were performed using a Hewlett-Packard 77,020 A ultrasound color system for M-mode, 2-dimensional, and Doppler examinations^32^. Examinations were performed by the same experienced cardiologist (Markku Ikäheimo) blinded to the patients’ clinical data. Left ventricular mass (LVM) was calculated using the formula by Troy et al.^33^, and the LVM index (LVMi) by dividing the difference between LVM values by body surface area. Standard and modern parameters including tissue Doppler–based measurements were determined according to the American Society of Echocardiography recommendations^32^. The manuscript does not include identifiable information or images of study subjects.

### Liver fat accumulation

Liver adiposity was assessed using liver-kidney contrast measured with ultrasonography (Toshiba SSA 270 ultrasound system, 5 MHz, Toshiba Corp., Tokyo, Japan) by an experienced radiologist (Markku Päivänsalo)^27^. The procedure was video-recorded and analyzed later. Hepatic steatosis severity was classified based on liver brightness: 0 (normal brightness, non-fatty liver), 1 (medium brightness, moderate lipid content) and 2 (clearly bright, severe lipid content). We compared subjects with normal liver brightness (group 0) to those with fatty liver (groups 1 and 2 combined)^34^. The manuscript does not include identifiable information or images.

### Outcome classification

Outcome classifications have been previously described^35^. Total mortality data were obtained from the Finnish Causes of Death Register, with an average follow-up time of over 26 years. CVD event and mortality data were collected after the end of 2021, averaging over 24 years of follow-up. CVDs and CVD mortality included major CHD events and stroke (excluding subarachnoid hemorrhage [SAH]). CHD as a cause of death included I20-I25, I46, R96, R98 (ICD-10/410-414, 798 (not 7980 A) (ICD-8/9)) as underlying or immediate causes, and I21 or I22 (ICD-10)/410 (ICD-8/9) as first to third contributing causes of death. Stroke (excluding SAH) included I61, I63 (not I636), I64 (ICD-10)/431, 4330 A, 4331 A, 4339 A, 4340 A, 4341 A, 4349 A, 436 (ICD-9)/431 (except 43101, 43191), 433, 434, 436 (ICD-8) as main or side diagnoses or contributing causes. Data on HF and CVD events were collected until the end of 2014 from the Hospital Discharge Register, with an average follow-up of over 20 years.

### Statistical methods

Prior to analysis, continuous variables were checked for skewness and kurtosis. NT-proANP levels were log-transformed before tertile division, which was done separately for the whole population, and for subjects without and with CVDs, CKD, DM or related medications. Normally distributed continuous variables are presented as mean (M) and standard deviation (SD). Non-normally distributed continuous variables are presented as median (Mdn) and interquartile range (IQR). Count data is presented as number of observations (n) and percentage (%). First, sex-specific NT-proANP tertiles were determined and then pooled for the analyses. For subpopulation analyses, the population was first divided based on morbidity and medications at baseline and only after that sex-specific NT-proANP tertiles were generated. For tertile comparisons, one-way ANOVA was used for normally distributed, Kruskal-Wallis’s test for non-normally distributed variables and Pearson’s Chi-square for categorical variables. The Bonferroni correction was used in post-hoc tests to adjust for multiple comparisons.

Logistic regression models were used to estimate the association of the NT-proANP tertiles with liver adiposity status at baseline. The initial regression model included all studied parameters that differed according to liver adiposity status. In the case of co-linear variables like DBP and SBP, only one was included in the model. Variables were then removed from the model based on statistical significance. The final model included only NT-proANP tertiles and variables with a significant effect on the outcome variable.

Cox proportional hazard models and Kaplan-Meier curves were used to estimate the association of the NT-proANP tertiles and longitudinal outcomes. For all outcomes, the follow-up time ended in case the outcome of interest was met or in case of death. The crude model included only the NT-proANP tertiles as an explanatory variable. Model 1 included sex and age. Model 2 included sex, age, smoking, alcohol consumption and previous diagnoses as additional explanatory variables. The explanatory variables were selected based on differences in descriptive statistics. Additional model in the supplementary material included smoking, alcohol consumption, BMI, 24 h SBP, eGFR, HOMA-IR, triglycerides and LDL cholesterol as additional explanatory variables and antihypertensive and acetyl salicylic acid (ASA) medications when applicable (Table S10). Subjects with missing data were omitted from the regression analyses. The low NT-proANP tertile (Tertile 1) was used as the reference group.

S.S and J.T had full access to all the data in the study and take responsibility for its integrity and data analysis.

## Results

### Characteristics of the study population in NT-proANP tertiles at baseline in all subjects and subjects without and with CVDs, CKD, DM or related medications

Baseline characteristics of the study population, and the two subpopulations comprising subjects without and with CVDs, CKD, DM or related medications are presented in Table 1 in NT-proANP tertiles. The highest NT-proANP tertile (Tertile 3) had the greatest variation in NT-proANP levels (295-2260 pmol/L, whole population analysis, Fig. S1). Subjects in Tertile 3 were older compared to subjects in the other NT-proANP tertiles (Table 1, Tertiles 1–2) in all three populations. Difference in smoking burden was only observed in subjects without CVDs, being the highest in Tertile 1. In whole population analysis and subjects with CVDs, Tertile 3 had the greatest prevalence of CVD morbidities including CAD, prior AMI and stroke or TIA at baseline, while no differences in the prevalence of diabetes or CKD were observed. For HTN, a difference was only observed between the NT-proANP tertiles in all subjects. BP medication use, especially beta-blocker use, was more frequent in Tertile 3 in all subjects, but not in the subjects with CVDs, whereas ASA using was most frequent in Tertile 3 in all subjects and subjects with CVDs. No difference in the use of lipid medication was observed between the Tertiles (Table 1).

**Table 1:** Characteristics of the study population in NT-proANP tertiles in all subjects and subjects without and with CVDs, CKD, DM or related medications. N; number of, M; mean, SD; standard deviation, Mdn; median, IQR; interquartile range, NT-proANP; N-terminal pro-atrial natriuretic peptide, Yrs; years, Wk; week, HTN; hypertension, CAD; coronary artery disease, AMI; acute myocardial infarction, TIA; transient ischemic attack, CKD; chronic kidney disease, DM; diabetes mellitus, BP; blood pressure, ASA; acetyl Salicylic acid. For tertile comparisons, one-way ANOVA was used for normally distributed, Kruskal–Wallis’s test for non-normally distributed variables and Pearson’s Chi-square for categorical variables. ¤*P* < 0.05 (Tertiles 1 and 2), #*P* < 0.05 (Tertiles 2 and 3) and §*P* < 0.05 (Tertiles 1 and 3) in post-hoc analysis.

All subjects	Tertile 1		Tertile 2		Tertile 3		
	n	M(SD)/Mdn(IQR)	n	M(SD)/Mdn(IQR)	n	M(SD)/Mdn(IQR)	*P*
NT-proANP (pmol/L)	345	151 (117–176)	353	247 (225–276)	346	390 (344–490)	< 0.001^¤#§^
Males, n (%)	345	172 (49.9)	353	175 (49.6)	346	173 (50.0)	0.99
Age (yrs)	345	49.7 (5.5)	353	51.0 (5.9)	346	53.0 (6.1)	< 0.001^¤#§^
Smoking (pack yrs)	345	4 (0–16)	353	1 (0–15)	346	0 (0–18)	0.33
Alcohol consumption (g/wk)	345	24 (3–84)	353	24 (2–86)	346	26 (1–79)	0.75
Diagnosis of HTN, n (%)	345	163 (47.2)	353	173 (49.0)	346	203 (58.7)	0.005^#§^
Diagnosis of CAD, n (%)	337	14 (4.2)	338	28 (8.3)	334	43 (12.9)	< 0.001^¤#^
Diagnosis of CKD, n (%)	345	9 (2.6)	353	14 (4.0)	346	19 (5.5)	0.15
Diagnosis of DM, n (%)	345	37 (10.7)	353	32 (9.1)	346	37 (10.7)	0.71
Prior AMI, n (%)	345	2 (0.6)	353	9 (2.5)	346	14 (4.0)	0.011^¤§^
Prior Stroke or TIA, n (%)	345	8 (2.3)	353	10 (2.8)	346	20 (5.8)	0.032^§^
Beta blocker users, n (%)	345	62 (18.0)	353	77 (21.8)	346	146 (42.2)	< 0.001^#§^
BP medication users, n (%)	345	164 (47.5)	353	172 (48.7)	346	205 (59.2)	0.003^#§^
Lipid medication users, n (%)	345	11 (3.2)	353	11 (3.1)	346	8 (2.3)	0.74
ASA users, n (%)	345	8 (2.3)	353	17 (4.8)	346	32 (9.2)	< 0.001^#§^
Subjects without CVDs							
NT-proANP (pmol/L)	138	154 (125–175)	136	226 (209–259)	136	358 (310–412)	< 0.001^¤#§^
Males, n (%)	138	68 (49.3)	136	67 (49.3)	136	67 (49.3)	1.00
Age (yrs)	138	49.1 (5.6)	136	50.6 (5.6)	136	51.9 (6.3)	< 0.001^§^
Smoking (pack yrs)	138	7 (0–18)	136	0 (0–11)	136	0 (0–14)	0.017^¤§^
Alcohol consumption (g/wk)	138	24 (6–89)	136	24 (2–74)	136	21 (2–76)	0.55
Subjects with CVDs							
NT-proANP (pmol/L)	213	149 (114–182)	210	261 (241–290)	211	429 (362–533)	< 0.001^¤#§^
Males, n (%)	213	107 (50.2)	210	105 (50.0)	211	106 (50.2)	0.99
Age (yrs)	213	50.1 (5.5)	210	51.8 (6.0)	211	53.4 (5.9)	< 0.001^¤#§^
Smoking (pack yrs)	213	1 (0–15)	210	2 (0–17)	211	1 (0–20)	0.96
Alcohol consumption* (g/wk)	213	24 (3–84)	210	28 (1–96)	211	27 (2–84)	0.89
Diagnosis of HTN, n (%)	213	182 (85.4)	210	181 (86.2)	211	176 (83.4)	0.71
Diagnosis of CAD, n (%)	213	22 (10.3)	210	46 (21.9)	211	52 (24.6)	< 0.001^¤§^
Diagnosis of CKD, n (%)	213	11 (5.2)	210	15 (7.1)	211	16 (7.6)	0.57
Diagnosis of DM, n (%)	213	39 (18.3)	210	34 (16.2)	211	33 (15.6)	0.74
Prior AMI, n (%)	213	3 (1.4)	210	9 (43)	211	13 (6.2)	0.04
Prior Stroke or TIA, n (%)	213	7 (3.3)	210	12 (5.7)	211	19 (9.0)	0.045
Beta blocker users, n (%)	213	72 (33.8)	210	76 (36.2)	211	137 (64.9)	< 0.001^#§^
BP medication users, n (%)	213	181 (85.0)	210	176 (83.8)	211	184 (87.2)	0.61
Lipid medication users, n (%)	213	11 (5.2)	210	11 (5.2)	211	8 (3.8)	0.73
ASA users, n (%)	213	10 (4.7)	210	17 (8.1)	211	30 (14.2)	0.002^§^

### Laboratory and clinical measurements of the study subjects in all subjects and subjects without and with CVDs, CKD, DM or related medications in NT-proANP tertiles

Laboratory and clinical measurements for all subjects and subjects without and with CVDs, CKD, DM or related medications in NT-proANP tertiles are presented in Table 2. Difference in BMI and leucocyte counts was only observed in subjects without CVDs between the NT-proANP tertiles, with Tertile 1 having the highest BMI accompanied with the highest leucocyte count. For eGFR, the lowest values were obserced in Tertile 3 in both all subjects and subjects with CVDs, CKD, DM or related medications. Out of glucose metabolism parameters, Tertile 1 had the highest fasting insulin levels accompanied with the highest HOMA-IR values in all subpopulations. Regarding lipid metabolism, differences according to NT-proANP tertiles were observed mostly in subjects without CVDs where Tertile 1 had the highest total cholesterol and triglyceride levels (Table 2).

**Table 2:** Clinical and laboratory measurements of the study subjects in all subjects, and subjects without and with CVDs, CKD, DM or related medications. N; number of, M; mean, SD; standard deviation, Mdn; median, IQR; interquartile range, BMI; body mass index, eGFR; estimated glomerular filtration rate, HOMA-IR; homeostatic model assessment for insulin resistance, HDL; high-density lipoprotein, LDL; low-density lipoprotein. For tertile comparisons, one-way ANOVA was used for normally distributed, Kruskal-Wallis’s test for non-normally distributed variables and Pearson’s Chi-square for categorical variables. ¤*P* < 0.05 (Tertiles 1 and 2), #*P* < 0.05 (Tertiles 2 and 3) and §*P* < 0.05 (Tertiles 1 and 3) in post-hoc analysis.

All subjects	Tertile 1		Tertile 2		Tertile 3		
	n	M(SD)/Mdn(IQR)	n	M(SD)/Mdn(IQR)	n	M(SD)/Mdn(IQR)	*P*
BMI (kg/m^2^)	345	28.0 (4.6)	353	27.5 (4.7)	346	27.6 (4.6)	0.41
Leucocytes (E9/L)	345	5.6 (4.8–6.9)	353	5.5 (4.8–6.6)	346	5.6 (4.7–6.6)	0.44
Hemoglobin (g/L)	345	143.5 (13.7)	353	142.4 (12.5)	346	140.9 (13.2)	0.030^§^
eGFR (mL/min/1.73m^3^)	344	87.2 (14.4)	353	84.8 (14.3)	345	81.1 (16.4)	< 0.001^#§^
Fasting glucose (mmol/L)	345	4.8 (1.7)	353	4.6 (1.2)	346	4.8 (1.4)	0.31
Fasting insulin (mmol/L)	345	11.4 (7.8–17.7)	353	10.6 (7.2–15.7)	346	10.0 (7.3–15.8)	0.037^§^
HOMA-IR	345	2.4 (1.5–3.8)	353	2.1 (1.3–3.2)	346	2.0 (1.4–3.5)	0.038^¤^
Total cholesterol (mmol/L)	345	5.8 (1.1)	353	5.7 (1.0)	346	5.6 (1.1)	0.37
Triglycerides (mmol/L)	345	1.4 (1.0–2.0)	353	1.3 (1.0–1.7)	346	1.3 (1.0–1.9)	0.057^¤^
HDL cholesterol (mmol/L)	345	1.3 (0.4)	353	1.4 (0.4)	346	1.4 (0.4)	0.15
LDL cholesterol (mmol/L)	345	3.6 (0.9)	353	3.5 (0.9)	346	3.5 (1.0)	0.80
Subjects without CVDs							
BMI (kg/m^2^)	138	27.1 (4.1)	136	25.7 (3.9)	136	25.3 (3.4)	< 0.001^¤§^
Leucocytes (E9/L)	138	5.5 (4.7–6.9)	136	5.2 (4.5–6.3)	136	5.2 (4.2–6.1)	0.012^¤^
Hemoglobin (g/L)	138	141.7 (14.8)	136	141.2 (12.2)	136	138.9 (11.7)	0.18
eGFR (mL/min/1.73m^3^)	137	88.4 (13.5)	136	87.1 (14.1)	135	85.8 (13.7)	0.29
Fasting glucose (mmol/l)	138	4.3 (4.1–4.6)	136	4.4 (4.0–4.7)	136	4.3 (4.0–4.5)	0.17
Fasting insulin (mmol/l)	138	10.1 (7.2–15.0)	136	8.6 (6.4–12.0)	136	8.1 (6.1–10.8)	< 0.001^§^
HOMA-IR	138	2.0 (1.4–2.8)	136	1.7 (1.2–2.4)	136	1.6 (1.1–2.1)	< 0.001^§^
Total cholesterol (mmol/L)	138	5.7 (1.1)	136	5.6 (0.9)	136	5.5 (0.9)	0.083
Triglycerides (mmol/L)	138	1.2 (0.9–1.7)	136	1.1 (0.9–1.4)	136	1.1 (0.8–1.4)	0.004^¤§^
HDL cholesterol (mmol/L)	138	1.4 (0.4)	136	1.4 (0.4)	136	1.5 (0.4)	0.056^§^
LDL cholesterol (mmol/L)	138	3.6 (1.0)	136	3.5 (1.8)	136	3.4 (0.9)	0.23
Subjects with CVDs							
BMI (kg/m2)	213	28.9 (4.8)	210	28.9 (5.0)	211	28.5 (4.5)	0.57
Leucocytes (E9/L)	213	5.7 (5.0–6.8)	210	5.8 (4.9–6.9)	211	5.8 (4.9–6.9)	0.69
Hemoglobin (g/L)	213	144.6 (13.1)	210	143.6 (12.2)	211	141.8 (13.9)	0.093
eGFR (mL/min/1.73 m^3^)	213	86.1 (15.0)	210	83.4 (14.8)	211	78.3 (16.8)	< 0.001^#§^
Fasting glucose (mmol/l)	213	4.5 (4.2–5.2)	210	4.5 (4.1–5.0)	211	4.5 (4.2–5.1)	0.27
Fasting insulin (mmol/l)	213	13.9 (9.3–20.4)	210	12.6 (8.2–18.5)	211	11.1 (8.1–17.7)	0.030^§^
HOMA-IR	213	3.0 (1.8–4.7)	210	2.5 (1.6–4.1)	211	2.3 (1.5–4.1)	0.031^§^
Total cholesterol (mmol/L)	213	5.8 (1.1)	210	5.8 (1.0)	211	5.7 (1.1)	0.73
Triglycerides (mmol/L)	213	1.6 (1.1–2.1)	210	1.4 (1.1–2.0)	211	1.4 (1.1–2.0)	0.36
HDL cholesterol (mmol/L)	213	1.3 (0.4)	210	1.3 (0.4)	211	1.3 (0.3)	0.30
LDL cholesterol (mmol/L)	213	3.6 (0.9)	210	3.6 (1.0)	211	3.6 (1.0)	0.92

### Echocardiographic and ambulatory blood pressure measurements in NT-proANP tertiles at baseline in all subjects and subjects without and with CVDs, CKD, DM or related medications

As the NT-proANP tertiles had distinct prevalences of CVDs and related medications such as beta-blockers, we next evaluated baseline echocardiographic and ambulatory blood pressure (BP) measurements in NT-proANP tertiles, presented in Table 3 and S3. In whole study population analysis, a J-shaped distribution was observed for most measures, with Tertile 3 having the longest/greatest measures and Tertile 2 having the shortest/smallest, respectively (Table 3).

**Table 3:** Echocardiographic measurements and ambulatory blood pressure measurements in NT-proANP tertiles in all subjects and subjects without and with CVDs, CKD, DM or related medications. N; number of, M; mean, SD; standard deviation, IVS; intraventricular septum, PVW; posterior ventricular wall, LVM; left ventricular mass, LVMi; left ventricular mass index, FS; fractional shortening, LVid; left ventricular internal diameter, LAd; left atrial diameter, E/A; early to atrial filling velocity, SBP; systolic blood pressure, DBP; diastolic blood pressure, HR; heart rate. For tertile comparisons, one-way ANOVA was used for normally distributed, Kruskal-Wallis’s test for non-normally distributed variables and Pearson’s Chi-square for categorical variables. ¤*P* < 0.05 (Tertiles 1 and 2), #*P* < 0.05 (Tertiles 2 and 3) and §*P* < 0.05 (Tertiles 1 and 3) in post-hoc analysis.

All subjects	Tertile 1		Tertile 2		Tertile 3		
	n	M(SD)	n	M(SD)	n	M(SD)	*P*
IVS (mm)	312	10.8 (2.0)	323	10.5 (2.1)	313	11.0 (2.4)	0.021^#^
PVW (mm)	311	10.1 (1.7)	322	10.0 (1.8)	311	10.4 (2.0)	0.012^#^
LVM (g)	311	244.8 (81.1)	322	239.3 (81.1)	311	258.3 (94.7)	0.060^#^
LVMi (g/m²)	311	128.9 (36.7)	322	126.9 (35.1)	311	137.9 (43.4)	0.007^#§^
FS (%)	311	34.7 (5.5)	322	35.3 (5.6)	311	34.8 (6.5)	0.35
LVid	311	51.4 (4.9)	322	51.6 (5.3)	311	51.9 (5.8)	0.60
LAd (mm)	293	38.4 (4.9)	309	38.8 (5.4)	293	39.9 (5.4)	0.002^#§^
E/A ratio	283	1.7 (0.5)	304	1.8 (0.6)	285	1.7 (0.6)	0.25
24 h SBP (mmHg)	291	131 (14)	317	128 (13)	295	130 (14)	0.037^¤§^
24 h DBP (mmHg)	291	83 (8)	317	81 (8)	295	80 (9)	0.002^¤§^
24 h HR (bpm)	291	73 (10)	317	70 (9)	295	68 (9)	< 0.001^¤#^
Subjects without CVDs							
IVS (mm)	122	10.3 (2.1)	129	10.0 (1.9)	128	9.9 (1.7)	0.19
PVW (mm)	122	9.7 (1.8)	129	9.5 (1.6)	128	9.4 (1.6)	0.48
LVM (g)	122	230.7 (89.1)	129	222.8 (77.0)	128	216.7 (70.0)	0.44
LVMi (g/m²)	122	123.5 (40.1)	129	120.0 (33.8)	128	119.4 (32.6)	0.75
FS (%)	122	33.9 (5.5)	129	35.1 (5.5)	128	34.7 (5.4)	0.1
LVid	122	51.4 (4.9)	129	51.6 (5.0)	128	51.2 (5.0)	0.84
LAd (mm)	129	37.3 (4.6)	130	37.7 (4.9)	132	37.6 (4.9)	0.83
E/A ratio	124	1.8 (0.5)	126	1.8 (0.6)	129	1.9 (0.6)	0.51
24 h SBP (mmHg)	115	129 (13)	120	125 (11)	120	125 (13)	0.042^¤^
24 h DBP (mmHg)	115	81 (8)	120	79 (7)	120	78 (8)	0.011^¤^
24 h HR (bpm)	115	74 (9)	120	70 (9)	120	70 (8)	< 0.001^¤#^
Subjects with CVDs							
IVS (mm)	192	11.1 (2.1)	193	11.2 (2.3)	184	11.6 (2.4)	0.10
PVW (mm)	191	10.4 (1.6)	192	10.5 (1.8)	182	10.9 (2.0)	0.023^§^
LVM (g)	191	251.3 (77.2)	192	260.5 (81.8)	182	279.4 (99.2)	0.006^§^
LVMi (g/m²)	191	130.9 (34.1)	192	136.1 (35.6)	182	147.8 (45.7)	< 0.001^§^
FS (%)	191	35.1 (5.4)	192	35.2 (5.8)	182	35.4 (6.9)	0.85
LVid (mm)	191	51.2 (5.3)	192	51.9 (5.3)	182	52.3 (6.3)	0.19
LAd (mm)	170	39.4 (5.2)	169	40.4 (5.2)	165	41.0 (5.4)	0.027^§^
E/A ratio	165	1.6 (0.5)	166	1.7 (0.5)	162	1.7 (0.7)	0.41
24 h SBP (mmHg)	181	133 (14)	191	132 (13)	176	132 (14)	0.76
24 h DBP (mmHg)	181	84 (8)	191	82 (8)	176	81 (9)	0.028^§^
24 h HR (bpm)	181	72 (10)	191	70 (10)	176	67 (10)	< 0.001^¤#§^

Clear differences in the measures were observed between the NT-proANP tertiles in the subpopulations based on CVDs, CKD, DM or related medications. In the subpopulation with CVDs, CKD, DM or related medications, Tertile 3 had the thickest posterior ventricular wall (PVW), the greatest LVM and LVMi, and the widest left atrial diameter (LAd). For LAd, an incremental increase was observed according to the NT-proANP tertiles. No differences were observed in intraventricular septum (IVS), fractional shortening (FS), left ventricular internal diameter (LVid), or early to atrial filling velocity (E/A) ratio between the Tertiles.

In the subpopulation without CVDs, CKD, DM or related medications the opposite was observed, as Tertile 3 had the shortest/smallest measures although no statistically significant differences were observed between the NT-proANP tertiles.

Regarding ambulatory BP measurements, a declining trend in 24 h SBP, 24 h DBP and 24 h HR was observed across all subpopulations. As an exception, 24 h SBP was U-shaped among the NT-proANP tertiles in all subjects. Additionally, no statistically significant difference was found in 24 h SBP in subjects with CVDs.

### NT-proANP levels differ but are not independently associated with liver adiposity

**Fig. 1:**
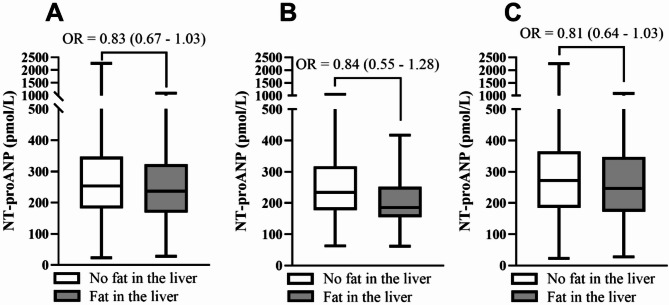
NT-proANP levels according to liver fat accumulation in the study population (**A**), subjects without (**B**) and with (**C**) CVDs, CKD, DM or related medications. The values are median (range). Odds ratio (OR) with 95% confidence interval (CI) is for NT-proANP levels from multivariable logistic regression.

As the NT-proANP tertiles had distinct levels of fasting insulin, insulin resistance scores and in the case of the subpopulation without CVDs, CKD, DM or related medications, BMI, suggesting insulin resistance and adiposity being negatively associated with NT-proANP levels, we next evaluated baseline NT-proANP levels according to liver adiposity status in the whole population and the two subpopulations, presented in Fig. 1. Regardless of subpopulation, subjects without liver adiposity had higher NT-proANP levels compared to subjects with liver adiposity (Fig. 1). The difference in NT-proANP levels appeared to be the largest in the population without CVDs, CKD, DM or related medications at baseline (Fig. 1B). However, in multivariable logistic regression models, the NT-proANP tertiles were not significantly associated with liver adiposity in the whole population nor either of the two subpopulations (Tables S4–S8).

### Prevalence of HF, CVD events, CVD mortality and total mortality during the 31-year follow-up in NT-proANP tertiles

Finally, we utilized the up-to 31 years of longitudinal data to evaluate the effect of NT-proANP tertiles for HF and CVD events and mortality rates in the whole study population and the two subpopulations in Cox regression models and Kaplan-Meier curves (Fig. S2, Tables 4, 5, 6 and 7, S8–9).

**Table 4:** Hazard ratios (HR) for heart failure in NT-proANP tertiles in all subjects and subjects without and with CVDs, CKD, DM or related medications. Cox regression models representing HR and 95% confidence intervals (CIs) with lower limits (CIL) and upper limit (CIU). NT-proANP tertile 1 = reference group. NT-proANP; N-terminal pro-atrial natriuretic peptide, CI; 95% confidence interval, CVD; cardiovascular disease, G; grams, Wk; week, HTN; hypertension, CAD; coronary artery disease, DM; diabetes mellitus, AMI; acute myocardial infarction, TIA; transient ischemic attack.

	All subjects	Subjects without CVDs	Subjects with CVDs
	HR (95% CI)	HR (95% CI)	HR (95% CI)
Crude Model			
NT-proANP tertile 1 (Low)	1	1	1
NT-proANP tertile 2	0.84 (0.43–1.84)	2.03 (0.18–22.42)	1.23 (0.62–2.43)
NT-proANP tertile 3 (High)	2.95 (1.62–5.31)	10.41 (1.33–81.34)	2.58 (1.42–4.70)
Model 1			
NT-proANP tertile 1 (Low)	1	1	1
NT-proANP tertile 2	0.76 (0.36–1.60)	1.69 (0.15–18.62)	1.08 (0.52–2.22)
NT-proANP tertile 3 (High)	2.26 (1.23–4.16)	6.43 (0.80–51.46)	1.71 (0.88–3.34)
Sex (female)	0.57 (0.35–0.94)	1.04 (0.34–3.13)	0.49 (0.28–0.86)
Age (years)	1.07 (1.03–1.12)	1.19 (1.05–1.34)	1.05 (1.00–1.10)
Model 2			
NT-proANP tertile 1 (Low)	1	-	1
NT-proANP tertile 2	0.69 (0.32–1.46)	-	0.79 (0.49–1.25)
NT-proANP tertile 3 (High)	1.96 (1.05–3.67)	-	1.45 (0.95–2.19)
Sex (female)	0.84 (0.48–1.49)	-	0.57 (0.38–0.85)
Age (years)	1.06 (1.01–1.11)	-	1.08 (1.05–1.12)
Smoking (pack-years)	1.01 (1.00–1.02)	-	1.02 (1.01–1.03)
Alcohol consumption (g/wk)	1.00 (1.00–1.00)	-	1.00 (1.00–1.00)
Diagnosis of HTN	1.28 (0.76–2.17)	-	1.10 (0.77–1.58)
Diagnosis of CAD	1.37 (0.67–2.84)	-	1.59 (0.94–2.68)
Diagnosis of CKD	2.04 (0.80–5.22)	-	1.23 (0.50–3.03)
Diagnosis of DM	1.47 (0.78–2.79)	-	3.27 (2.16–4.95)
Prior AMI	2.29 (0.85–6.20)	-	1.78 (0.83–3.79)
Prior stroke or TIA	0.77 (0.27–2.20)	-	1.47 (0.75–2.87)

Regarding HF events, in whole population analysis, the highest NT-proANP tertile was at a slightly higher risk for HF events in all three models with HR ranging from 1.96 (1.05–3.67) to 2.95 (1.62–5.31) depending on the model (Table 4, Table S9). In subjects without CVDs, CKD, DM or related medications at baseline, the highest NT-proANP was at higher risk for HF in the crude model, but not in the adjusted models (Table 4, Table S9). There were only 13 HF events in this subpopulation during the follow-up period (Table S8). In subjects with CVDs, CKD, DM or related medications at baseline, the highest NT-proANP was at higher risk for HF in the crude model, but not in the adjusted models (Table 4, Table S9).

Regarding CVD events, in whole population analysis, the highest NT-proANP tertile was at a slightly higher risk for CVD events only in the crude model (Table 5, Table S9). In subjects with or without CVDs, CKD, DM or related medications at baseline, the NT-proANP tertiles were not associated with CVD events in any of the models (Table 5, S9).

**Table 5:** Hazard ratios (HR) for CVD events in NT-proANP tertiles in all subjects and subjects without and with CVDs, CKD, DM or related medications. Cox regression models representing HR and 95% confidence intervals (CIs) with lower limits (CIL) and upper limit (CIU). NT-proANP tertile 1 = reference group. NT-proANP; N-terminal pro-atrial natriuretic peptide, CI; 95% confidence interval, CVD; cardiovascular disease, G; grams, Wk; week, HTN; hypertension, CAD; coronary artery disease, DM; diabetes mellitus, AMI; acute myocardial infarction, TIA; transient ischemic attack.

	All subjects	Subjects without CVDs	Subjects with CVDs
	HR (95% CI)	HR (95% CI)	HR (95% CI)
Crude model			
NT-proANP tertile 1 (Low)	1	1	1
NT-proANP tertile 2	1.11 (0.83–1.54)	0.61 (0.32–1.19)	1.09 (0.80–1.48)
NT-proANP tertile 3 (High)	1.53 (1.12–2.01)	1.21 (0.69–2.13)	1.32 (0.98–1.78)
Model 1			
NT-proANP tertile 1 (Low)	1	1	1
NT-proANP tertile 2	0.98 (0.71–1.35)	0.57 (0.29–1.11)	1.18 (0.82–1.69)
NT-proANP tertile 3 (High)	1.16 (0.84–1.58)	0.96 (0.54–1.70)	1.16 (0.80–1.67)
Sex (female)	0.40 (0.30–0.52)	0.33 (0.19–0.58)	0.42 (0.31–0.57)
Age (years)	1.07 (1.04–1.09)	1.08 (1.03–1.13)	1.06 (1.03–1.08)
Model 2			
NT-proANP tertile 1 (Low)	1	-	1
NT-proANP tertile 2	0.93 (0.67–1.28)	-	0.99 (0.77–1.27)
NT-proANP tertile 3 (High)	1.06 (0.77–1.46)	-	1.16 (0.90–1.47)
Sex (female)	0.48 (0.36–0.65)	-	0.71 (0.57–0.89)
Age (years)	1.05 (1.03–1.08)	-	1.10 (1.08–1.12)
Smoking (pack-years)	1.01 (1.01–1.02)	-	1.02 (1.01–1.03)
Alcohol consumption (g/wk)	1.00 (1.00–1.00)	-	1.00 (1.00–1.00)
Diagnosis of HTN	1.38 (1.06–1.80)	-	1.06 (0.86–1.29)
Diagnosis of CAD	1.84 (1.27–2.68)	-	1.37 (1.01–1.85)
Diagnosis of CKD	1.54 (0.85–2.78)	-	1.89 (1.24–2.87)
Diagnosis of DM	1.82 (1.30–2.55)	-	2.30 (1.76–3.00)
Prior AMI	1.76 (0.98–3.15)	-	1.12 (0.65–1.95)
Prior stroke or TIA	1.30 (0.78–2.17)	-	1.26 (0.82–1.94)

When analyzing CVD mortality, in whole population analysis, the highest NT-proANP tertile was at a slightly higher risk for CVD mortality in the crude model and in the additional model laboratory measure-adjusted model (Table 6, S9). In subjects without CVDs, CKD, DM or related medications at baseline, the NT-proANP tertiles were associated with CVD events only in the additional model HR 5.12 (1.62–16.43) (Table S9). Subjects with a CVD-related death in this subpopulation were more often males (67.6%), older (4.1 years older) and smoked more (12 pack-years more) compared to those without a CVD-related death (Table S10). In subjects with CVDs, CKD, DM or related medications at baseline, the NT-proANP tertiles were associated with CVD mortality in the crude model HR 1.87 (1.25–2.78) and in the additional model HR 2.15 (1.34–3.46) (Table 6, Table S9).

**Table 6:** Hazard ratios (HR) for CVD mortality in NT-proANP tertiles in all subjects and subjects without and with CVDs, CKD, DM or related medications. Cox regression models representing HR and 95% confidence intervals (CIs) with lower limits (CIL) and upper limit (CIU). NT-proANP tertile 1 = reference group. NT-proANP; N-terminal pro-atrial natriuretic peptide, CI; 95% confidence interval, CVD; cardiovascular disease, G; grams, Wk; week, HTN; hypertension, CAD; coronary artery disease, DM; diabetes mellitus, AMI; acute myocardial infarction, TIA; transient ischemic attack.

	All subjects	Subjects without CVDs	Subjects with CVDs
	HR (95% CI)	HR (95% CI)	HR (95% CI)
Crude model			
NT-proANP tertile 1 (low)	1	1	1
NT-proANP tertile 2	1.01 (0.63–1.64)	0.75 (0.30–1.87)	0.94 (0.60–1.49)
NT-proANP tertile 3 (high)	2.04 (1.32–2.91)	1.69 (0.80–3.58)	1.87 (1.25–2.78)
Model 1			
NT-proANP tertile 1 (low)	1	1	1
NT-proANP tertile 2	0.86 (0.54–1.36)	0.65 (0.26–1.62)	0.90 (0.53–1.54)
NT-proANP tertile 3 (high)	1.41 (0.93–2.15)	1.14 (0.53–2.46)	1.58 (0.97–2.56)
Sex (female)	0.36 (0.25–0.51)	0.38 (0.19–0.76)	0.35 (0.23–0.54)
Age (years)	1.10 (1.06–1.13)	1.14 (1.07–1.21)	1.07 (1.04–1.11)
Model 2			
NT-proANP tertile 1 (low)	1	–	1
NT-proANP tertile 2	0.86 (0.54–1.36)	–	0.99 (0.49–1.98)
NT-proANP tertile 3 (high)	1.36 (0.89–2.11)	–	1.85 (0.99–3.46)
Sex (female)	0.57 (0.38–0.85)	–	0.82 (0.47–1.45)
Age (years)	1.08 (1.05–1.12)	–	1.07 (1.02–1.12)
Smoking (pack-years)	1.02 (1.01–1.03)	–	1.01 (1.00–1.02)
Alcohol consumption (g/wk)	1.00 (1.00–1.00)	–	1.00 (1.00–1.00)
Diagnosis of HTN	1.07 (0.74–1.53)	–	1.41 (0.84–2.38)
Diagnosis of CAD	1.56 (0.93–2.62)	–	1.32 (0.64–2.75)
Diagnosis of CKD	1.22 (0.49–3.01)	–	2.21 (0.86–5.66)
Diagnosis of DM	3.20 (2.12–4.85)	–	1.51 (0.80–2.86)
Prior AMI	1.62 (0.76–3.46)	–	2.30 (0.85–6.24)
Prior stroke or TIA	1.51 (0.78–2.94)	–	0.76 (0.26–2.21)

**Table 7:** Hazard ratios (HR) for total mortality in NT-proANP tertiles in all subjects and subjects without and with CVDs, CKD, DM or related medications. Cox regression models representing HR and 95% confidence intervals (CIs) with lower limits (CIL) and upper limit (CIU). NT-proANP tertile 1 = reference group. NT-proANP; N-terminal pro-atrial natriuretic peptide, CI; 95% confidence interval, CVD; cardiovascular disease, G; grams, Wk; week, HTN; hypertension, CAD; coronary artery disease, DM; diabetes mellitus, AMI; acute myocardial infarction, TIA; transient ischemic attack.

	All subjects	Subjects without CVDs	Subjects with CVDs
	HR (95% CI)	HR (95% CI)	HR (95% CI)
Crude Model			
NT-proANP tertile 1 (Low)	1	1	1
NT-proANP tertile 2	1.12 (0.94–1.45)	1.05 (0.69–1.58)	1.12 (0.88–1.44)
NT-proANP tertile 3 (High)	1.53 (1.22–1.93)	1.11 (0.74–1.67)	1.47 (1.16–1.86)
Model 1			
NT-proANP tertile 1 (Low)	1	1	1
NT-proANP tertile 2	0.93 (0.73–1.20)	0.92 (0.60–1.39)	1.00 (0.73–1.36)
NT-proANP tertile 3 (High)	1.09 (0.85–1.39)	0.80 (0.52–1.22)	1.28 (0.95–1.73)
Sex (female)	0.52 (0.42–0.63)	0.40 (0.28–0.57)	0.59 (0.46–0.75)
Age (years)	1.10 (1.08–1.12)	1.12 (1.08–1.15)	1.09 (1.07–1.12)
Model 2			
NT-proANP tertile 1 (low)	1	-	1
NT-proANP tertile 2	0.94 (0.73–1.21)	-	0.94 (0.68–1.28)
NT-proANP tertile 3 (high)	1.10 (0.86–1.41)	-	1.09 (0.80–1.49)
Sex (female)	0.71 (0.57–0.89)	-	0.48 (0.36–0.65)
Age (years)	1.1 (1.08–1.12)	-	1.05 (1.03–1.08)
Smoking (pack-years)	1.02 (1.01–1.03)	-	1.01 (1.01–1.02)
Alcohol consumption (g/wk)	1.00 (1.00–1.00)	-	1.00 (1.00–1.00)
Diagnosis of HTN	1.04 (0.85–1.28)	-	1.39 (1.07–1.80)
Diagnosis of CAD	1.37 (1.01–1.85)	-	1.84 (1.27–2.69)
Diagnosis of CKD	1.87 (1.23–2.84)	-	1.53 (0.85–2.76)
Diagnosis of DM	2.29 (1.79–2.99)	-	1.84 (1.31–2.57)
Prior AMI	1.11 (0.64–1.92)	-	1.80 (1.00–3.23)
Prior stroke or TIA	1.26 (0.82–1.94)	-	1.29 (0.77–2.16)

For total mortality, in whole population analysis, the NT-proANP tertiles were associated with total mortality only in the crude model HR 1.53 (1.22–1.93) (Table 7). In subjects without CVDs, CKD, DM or related medications at baseline the NT-proANP tertiles were not associated with total mortality in any of the models (Table 7, Table S9). In subjects with CVDs, CKD, DM or related medications at baseline, the NT-proANP tertiles were associated with total mortality only in the crude model HR 1.47 (1.16–1.86) (Table 7, S9).

## Discussion

### Main results and their relation to previous findings

In subjects without CVDs, DM or CKD at baseline, lower NT-proANP levels were cross-sectionally associated with an overall adverse metabolic profile but had little-to-no predictive value for longitudinal outcomes including HF and CVD events and mortality rates. In contrast, among subjects with CVDs, DM or CKD at baseline, higher NT-proANP levels were predictive for HF events and CVD mortality in longitudinal analysis. The current data highlights the significance of addressing baseline morbidity when evaluating NT-proANP levels longitudinally.

Higher NT-proANP levels have previously been associated with older age^36^, female sex^36^, beta blocker use^37^ and adverse kidney function^38^, as also observed here. In various study populations with varying CVD prevalence, higher NT-proANP levels have been associated with several CVD-related outcomes and total mortality. Here, in all cohort subjects the high NT-proANP tertile had higher prevalence of HTN, CAD, prior AMI, stroke and TIA at baseline and higher counts of BP medication and ASA users.

Counterintuitively to having the lowest CVD prevalence, the low NT-proANP tertile had an adverse metabolic profile, especially evident in the subpopulation without CVDs, CKD, DM or related medications. In these subjects, the low NT-proANP tertile had the highest BMI, the highest fasting insulin and triglyceride levels and the highest BP values. In a study of some 3000 subjects without CVDs but not excluding hypertension, NP levels were negatively associated with insulin resistance and other components of metabolic syndrome, except for hypertension^16^. The results also align with previous publications suggesting NT-proANP levels are negatively associated with hyperinsulinemia^16,39^ and obesity^40^. The associations observed here were more prominent in subjects without CVDs, CKD, DM or related medications and in females, who had lower prevalence of CVDs than males, perhaps suggesting CVDs negate the associations between NT-proANP and metabolic factors.

Lower levels of NT-proANP were detected in subjects with liver adiposity at baseline, like previously observed for NT-proBNP levels in healthy subjects^41^. This could be explained by the greater insulin resistance and adiposity present here in the low NT-proANP tertile, as insulin acts as lipid storing hormone in the liver^42^, and lower NT-proANP levels have been associated with obesity^39,40^ and insulin resistance^39^, both causal for liver adiposity. However, NT-proANP levels did not individually explain liver adiposity in multivariable regression models, suggesting no direct link between NT-proANP and liver adiposity.

The relationship between NPs and HTN remains undisclosed. Among all cohort subjects HTN and BP medication use, especially beta-blocker use, was more frequent in the high NT-proANP tertile. Beta-blocker treatment, often accompanied with cardiac diseases and hypertension, stimulates NP secretion^37,43,44^. In a previous study of some 3000 subjects without CVDs but not excluding hypertensive patients, NP levels were not associated with HTN^16^. Here, although the absolute differences in BP and HR were small, the low NT-proANP tertile had the highest BP values in subjects without CVDs, CKD, DM or related medications, also excluding HTN. Also, obesity is associated with low circulating NT-proANP, which may contribute to higher BP in subjects with lower NT-proANP levels^40^. Further, evidence exists that genetic variants of genes coding precursors for both ANP and BNP lead to higher circulating NP levels, which may contribute to higher BP in subjects with lower NP levels^45^. All in all, the association between BP and NT-proANP appears complex, and may differ between studied populations, study setup, and the peptide studied^46^.

Several interesting differences were observed in echocardiographic measures according to NT-proANP tertiles. In the subpopulation with CVDs, CKD, DM or related medications greater LAd and LVMi, signs of left atrial (LA) and left ventricular (LV) enlargement, often caused by conditions characterized by diastolic dysfunction and volume overload such as AF, left ventricular hypertrophy, and systemic hypertension, were observed according to the NT-proANP tertiles. However, in the subpopulation without CVDs, CKD, DM or related medications, the associations were not detectable. NT-proANP levels have been previously associated with LA dimensions^47^ and LA volume index has been associated with NT-proANP levels after AMI^48^. Overall, as no associations were detected in the subpopulation without CVDs, CKD, DM or related medication, the results suggest that the morphological differences in echocardiographic measures between the NT-proANP tertiles are mediated by conditions that cause both morphological changes to the heart and elevate NT-proANP levels, such as CVDs.

In the current follow-up period of up to 31 years, NT-proANP levels at middle-age predicted HF, as previously shown in shorter timeframes^8–10^, however, the population characteristics of previous studies and the current one differ drastically^16^. In a cohort of subjects with systolic HF, higher levels of ANP were associated with restrictive filling pattern and more severe HF^49^. In a cohort of 403 subjects post-AMI, elevated levels of NT-proANP were detected in subjects with HF^49^. In 3346 subjects without HF but not excluding other CVDs, higher levels of NT-proANP were associated with HF events during a mean follow-up of 5.2 years^8^. Here the HR for HF was the highest in subjects with CVDs, CKD, DM or related medications at baseline compared to whole cohort analysis and subjects without CVDs, CKD, DM or related medications at baseline, where the HR did not reach statistical significance. Overall, the results suggest that NT-proANP levels have predictive value for HF events especially in subjects with CVDs, CKD, DM or related medications.

NT-proANP levels have been associated with CVD events and CVD-related mortality in various setups. In 3346 subjects without HF but not excluding other CVDs, higher levels of NT-proANP were associated with CVD events, stroke and TIA during a mean follow-up of 5.2 years^8^. In 905 Finnish men (age 46–65 years) with various CVDs, NT-proANP levels were predictive of CVD deaths^10^ and in 131 subjects post-AMI, NT-proANP predicted CVD mortality and was associated with but did not provide additional predictive value over left ventricular ejection fraction^5^. Here, although the low NT-proANP tertile seemed to have the least favorable metabolic profile at baseline, having for example the highest BP, highest BMI and fasting insulin, the high NT-proANP tertile was predictive for CVD mortality in whole cohort analysis and in subjects with or without CVDs, CKD, DM or related medications at baseline. However, as the adverse metabolic profile observed in the low NT-proANP tertile did not translate to an increased risk longitudinally, the result is likely explained by the positive association of NT-proANP levels with older age, which outweighs the negative associations between NT-proANP levels and metabolic factors. Altogether, the mortality data suggests that higher NT-proANP levels at middle-age, mediated by older age, have prognostic value for CVD-related mortality, especially in populations with CVDs.

NT-proANP levels have been associated with total mortality in various setups. In a cohort of 403 subjects post-AMI, NT-proANP predicted mortality after 30 days^13^. In a community-based cohort of 2042 subjects, excluding patients with severe HF and CKD but not excluding subjects with other CVDs, NT-proANP had significant predictive value for mortality during a 9 year follow-up^9^. In a cohort of 85-year-olds, NT-proANP levels were predictive for 5-year mortality in both subjects without and with CVDs^12^. In contrast to previously observed higher total mortality rates in subjects with the highest NT-proANP levels^8–10^, no association with total mortality rate according to NT-proANP tertiles was observed here. As the HRs for total mortality were the highest in the high NT-proANP tertile and in subjects with CVDs, CKD, DM or related medications at baseline, and the lowest in subjects without CVDs, CKD, DM or related medications at baseline, and NT-proANP levels had predictive value for CVD mortality, the results suggest that NT-proANP levels are inconsequential for deaths not related to CVDs.

### Validity issues

The effect of confounders such as sex and baseline CVDs were accounted for by means of restriction to specific subpopulations and multivariate models. Tertile division was done separately for all subpopulations before pooling the tertiles together. Nevertheless, it is possible for some residual confounding to exist. Use of suggestive measures such as smoking can lead to discrepancies in the data. Also, some outcome parameters analyzed are linked (such as BP values and medications) which might lead to difficulties in data interpretation. Being aware of these challenges and analyzing not just individual parameters but also key clinical outcomes allow for more reliable conclusions to be drawn from our results.

There are limitations in the study population. The follow-up time is long, and we use a single measure of NT-proANP, thus an interaction with time cannot be overruled. Due to the setup of the original OPERA study, half of the subjects were hypertensive at baseline, thus the results do not translate to the general population directly. We lack the diagnosis of HF at baseline. However, as all BP medication users and co-morbidities of HF such as CAD were controlled for, it is likely that at least previously diagnosed HF were also controlled. As baseline echocardiography measurements were done already in the 1990s, we lack some of the more modern measures. For the same reason, some key medications that are nowadays stable in the treatment of CVDs such as sodium-glucose cotransporter-2 (SGLT2) inhibitors or angiotensin receptor/neprilysin inhibitor (ARNI) are not present in the current study, which decreases comparability to populations treated according to current guidelines, but also restricts the possible confounding effects caused by such treatments.

### Implications

The cross-sectional results can be interpreted as suggesting that high NT-proANP levels in middle-aged subjects are highly dependent on population characteristics. Higher levels are observed in females and NT-proANP levels increase with age. In subjects without CVDs, CKD, DM or related medications lower levels are associated with adverse metabolic traits, for example the highest BP, highest BMI and fasting insulin. In subjects with CVDs, CKD, DM or related medications high levels are indicative of CVDs and structural changes in echocardiography.

Longitudinally, NT-proANP levels at middle-age have some potential in the prediction of HF events over a 20 + year follow-up period and CVD mortality over an up to 31-year follow-up period. The usefulness of NT-proANP seems to be the highest in subjects with CVDs, also emphasized by the lack of increased risk in total mortality according to NT-proANP tertiles. Moreover, associations between NT-proANP and echocardiographic measures are only observed in subjects with CVDs, CKD, DM or related medications at baseline. The cross-sectional negative associations with insulin resistance and adiposity appear inconsequential as they neither translate to an increased risk for liver adiposity at baseline, nor to increased risk for the longitudinal outcomes. All in all, the data suggests that NT-proANP levels are elevated in subjects with CVDs where cardiac morphology is affected, and their predictive value is likely a reflection of the predictive value of these conditions.

## Conclusions

The cross-sectional and longitudinal associations of NT-proANP levels are highly dependent on population characteristics such as age and CVDs. In subjects with CVDs NT-proANP levels predict HF events and CVD mortality, while in subjects without CVDs, NT-proANP levels have less value in a longitudinal setup.

## Electronic supplementary material

Below is the link to the electronic supplementary material.

Supplementary Material 1

## Data Availability

Data is available upon reasonable request by contacting the corresponding author, joona.tapio@oulu.fi.
