# Supplementary material for: Prognostic value of NT-proANP levels on major cardiovascular outcomes in a 31-year follow-up study depends on baseline morbidity
Source: Sci Rep. 2025 May 28;15:18660. doi: 10.1038/s41598-025-03819-6 (PMC12119872; doi:10.1038/s41598-025-03819-6)
Supplement: Supplementary file 1 — Supplementary Material 1 [file 41598_2025_3819_MOESM1_ESM.docx]

**Prognostic value of NT-proANP levels on major cardiovascular outcomes in a 31-year follow-up study depends on baseline morbidity**

Samuli Sakko; B.M^1^, Juha Perkiömäki; MD, PhD^2^, Antti Ylitalo; MD, PhD^3^, Heikki Huikuri; MD, PhD^2^, Olavi Ukkola; MD, PhD^2^, Peppi Koivunen; MD, PhD^1^, Joona Tapio; MD, PhD^1*^

^1^Biocenter Oulu and Faculty of Biochemistry and Molecular Medicine, Research Unit for Extracellular matrix and hypoxia, University of Oulu, P.O. Box 5400, FIN-90014 Oulu, Finland.

^2^Research Unit of Biomedicine and Internal Medicine, Medical Research Center Oulu, University of Oulu and Oulu University Hospital, Oulu, Finland.

^3^Turku University Hospital, Heart Center, Turku, Finland and University of Turku, Turku, Finland

**Short title: NT-proANP and cardiometabolism**

**Corresponding author:** Joona Tapio, [joona.tapio@oulu.fi](mailto:joona.tapio@oulu.fi)

P.O. Box 5400 (Aapistie 7 C), FI-90014 University of Oulu, Finland

Total word count:

**Supplemental material**

**Table S1. Characteristics of the males and females of the study population in NT-proANP tertiles at baseline.** n; number of, M; Mean, SD; Standard deviation, Mdn; Median, IQR; interquartile range, NT-proANP; N-terminal pro-atrial natriuretic peptide, yrs; years, g/wk; grams per week, HTN; hypertension, CAD; coronary artery disease, AMI; acute myocardial infarction, TIA; transient ischemic attack, CKD; chronic kidney disease, DM; diabetes mellitus, BP; blood pressure, ASA; acetyl salicylic acid. For tertile comparisons, one-way ANOVA was used for normally distributed, Kruskal-Wallis’s test for non-normally distributed variables and Pearson’s Chi-square for categorical variables.

|  | Tertile 1 | | Tertile 2 | | Tertile 3 | |  |
| --- | --- | --- | --- | --- | --- | --- | --- |
| **Males** | n | M(SD)/Mdn(IQR) | n | M(SD)/Mdn(IQR) | n | M(SD)/Mdn(IQR) | *P* |
| NT-proANP (pmol/L) | 172 | 162 (131 - 181) | 175 | 238 (218 - 262) | 173 | 373 (335 - 455) | <0.001 |
| Age (yrs) | 172 | 48.5 (5.6) | 175 | 50.4 (5.9) | 173 | 53.2 (5.5) | <0.001 |
| Smoking (pack yrs) | 172 | 12 (0 - 22) | 175 | 10 (0 - 23) | 173 | 12 (0 - 29) | 0.42 |
| Alcohol consumption (g/wk) | 172 | 56 (12 - 133) | 175 | 72 (12 - 144) | 173 | 65 (15 - 139) | 0.73 |
| Diagnosis of HTN, n (%) | 172 | 64 (37.2) | 175 | 96 (54.9) | 173 | 108 (62.4) | <0.001 |
| Diagnosis of CAD, n (%) | 171 | 4 (2.3) | 169 | 13 (7.7) | 168 | 26 (15.5) | <0.001 |
| Diagnosis of CKD, n (%) | 172 | 3 (1.7) | 175 | 6 (3.4) | 173 | 7 (4.0) | 0.44 |
| Diagnosis of DM, n (%) | 172 | 18 (10.5) | 175 | 18 (10.3) | 173 | 22 (12.7) | 0.73 |
| Prior AMI, n (%) | 172 | 1 (0.6) | 175 | 9 (5.1) | 173 | 11 (6.4) | 0.016 |
| Prior Stroke or TIA, n (%) | 172 | 6 (3.5) | 175 | 5 (2.9) | 173 | 11 (6.4) | 0.23 |
| Beta blocker users, n (%) | 172 | 26 (15.1) | 175 | 50 (28.6) | 173 | 75 (43.4) | <0.001 |
| BP medication users, n (%) | 172 | 67 (39.0) | 175 | 94 (53.7) | 173 | 104 (60.1) | <0.001 |
| Lipid medication users, n (%) | 172 | 6 (3.5) | 175 | 7 (4.0) | 173 | 6 (3.5) | 0.96 |
| ASA users, n (%) | 172 | 3 (1.7) | 175 | 11 (6.3) | 173 | 23 (13.3) | <0.001 |
| **Females** |  |  |  |  |  |  |  |
| NT-proANP (pmol/L) | 173 | 141 (110 - 170) | 178 | 258 (234 - 285) | 173 | 415 (351 - 518) | <0.001 |
| Age (yrs) | 173 | 50.9 (5.1) | 178 | 51.6 (5.8) | 173 | 52.8 (6.6) | 0.009 |
| Smoking (pack yrs) | 173 | 0 (0 - 6) | 178 | 0 (0 - 5) | 173 | 0 (0 - 1) | 0.054 |
| Alcohol consumption (g/wk) | 173 | 12 (1 - 37) | 178 | 11 (1 - 33) | 173 | 6 (0 - 32) | 0.074 |
| Diagnosis of HTN, n (%) | 173 | 99 (57.2) | 178 | 77 (43.3) | 173 | 95 (54.9) | 0.019 |
| Diagnosis of CAD, n (%) | 166 | 10 (6.0) | 169 | 15 (8.9) | 166 | 17 (10.2) | 0.37 |
| Diagnosis of CKD, n (%) | 173 | 6 (3.5) | 178 | 8 (4.5) | 173 | 12 (6.9) | 0.31 |
| Diagnosis of DM, n (%) | 173 | 19 (11.0) | 178 | 14 (7.9) | 173 | 15 (8.7) | 0.58 |
| Prior AMI, n (%) | 173 | 1 (0.6) | 178 | 0 (0.0) | 173 | 3 (1.7) | 0.16 |
| Prior Stroke or TIA, n (%) | 173 | 2 (1.2) | 178 | 5 (2.8) | 173 | 9 (5.2) | 0.089 |
| Beta blocker users, n (%) | 173 | 36 (20.8) | 178 | 27 (15.2) | 173 | 71 (41.0) | <0.001 |
| BP medication users, n (%) | 173 | 97 (56.1) | 178 | 78 (43.8) | 173 | 101 (58.4) | 0.013 |
| Lipid medication users, n (%) | 173 | 5 (2.9) | 178 | 4 (2.2) | 173 | 2 (1.2) | 0.52 |
| ASA users, n (%) | 173 | 5 (2.9) | 178 | 6 (3.4) | 173 | 9 (5.2) | 0.50 |

**Table S2. Clinical and laboratory measurements of the males and females of the study population in NT-proANP tertiles at baseline.** n; number of, M; Mean, SD; Standard deviation, Mdn; Median, IQR; interquartile range, NT-proANP; N-terminal pro-atrial natriuretic peptide, BMI; body mass index, eGFR; estimated glomerular filtration rate, HOMA-IR; homeostatic model assessment for insulin resistance, HDL; high-density lipoprotein, LDL; low-density lipoprotein. For tertile comparisons, one-way ANOVA was used for normally distributed, Kruskal-Wallis’s test for non-normally distributed variables and Pearson’s Chi-square for categorical variables.

|  | Tertile 1 | | Tertile 2 | | Tertile 3 | |  |
| --- | --- | --- | --- | --- | --- | --- | --- |
| **Males** | n | M(SD)/Mdn(IQR) | n | M(SD)/Mdn(IQR) | n | M(SD)/Mdn(IQR) | *P* |
| BMI (kg/m^2^) | 172 | 27.7 (4.0) | 175 | 28.0 (4.4) | 173 | 28.1 (4.2) | 0.72 |
| Leucocytes (E9/L) | 172 | 5.8 (5.0 - 7.1) | 175 | 5.7 (4.8 - 6.9) | 173 | 5.9 (5.2 - 6.9) | 0.55 |
| Hemoglobin (g/L) | 172 | 152.7 (9.5) | 175 | 150.7 (9.7) | 173 | 148.9 (9.5) | 0.001 |
| eGFR (mL/min/1.73m^3^) | 172 | 92.7 (12.5) | 175 | 89.1 (14.1) | 173 | 85.1 (14.7) | <0.001 |
| Fasting glucose (mmol/L) | 172 | 4.8 (1.4) | 175 | 4.8 (1.5) | 173 | 5.0 (1.8) | 0.73 |
| Fasting insulin (mmol/L) | 172 | 12.5 (9.3 - 19.2) | 175 | 12.4 (8.6 - 19.0) | 173 | 11.9 (8.1 - 17.5) | 0.29 |
| HOMA-IR | 172 | 2.5 (1.8 - 3.9) | 175 | 2.5 (1.7 - 4.0) | 173 | 2.3 (1.6 - 4.0) | 0.57 |
| Total cholesterol (mmol/L) | 172 | 5.8 (1.1) | 175 | 5.8 (0.9) | 173 | 5.7 (1.1) | 0.43 |
| Triglycerides (mmol/L) | 172 | 1.5 (1.1 - 2.1) | 175 | 1.4 (1.1 - 2.0) | 173 | 1.5 (1.1 - 2.2) | 0.55 |
| HDL cholesterol (mmol/L) | 172 | 1.2 (0.3) | 175 | 1.2 (0.3) | 173 | 1.2 (0.3) | 0.055 |
| LDL cholesterol (mmol/L) | 172 | 3.7 (0.9) | 175 | 3.7 (0.9) | 173 | 3.6 (1.0) | 0.23 |
| **Females** |  |  |  |  |  |  |  |
| BMI (kg/m^2^) | 173 | 28.2 (5.1) | 178 | 27.1 (5.0) | 173 | 27.1 (4.8) | 0.059 |
| Leucocytes (E9/L) | 173 | 5.5 (4.7 - 6.7) | 178 | 5.4 (4.7 - 6.4) | 173 | 5.1 (4.4 - 6.1) | 0.062 |
| Hemoglobin (g/L) | 173 | 134.4 (10.8) | 178 | 134.3 (9.2) | 173 | 132.9 (11.4) | 0.31 |
| eGFR (mL/min/1.73m^3^) | 172 | 81.7 (14.2) | 178 | 80.5 (13.1) | 172 | 77.2 (17.0) | 0.014 |
| Fasting glucose (mmol/L) | 173 | 4.9 (2.0) | 178 | 4.4 (0.7) | 173 | 4.6 (1.0) | 0.12 |
| Fasting insulin (mmol/L) | 173 | 10.2 (6.9 - 16.6) | 178 | 8.8 (6.2 - 13.2) | 173 | 8.8 (6.3 - 13.3) | 0.047 |
| HOMA-IR | 173 | 1.9 (1.3 - 3.7) | 178 | 1.7 (1.2 - 2.6) | 173 | 1.8 (1.2 - 2.7) | 0.03 |
| Total cholesterol (mmol/L) | 173 | 5.7 (1.1) | 178 | 5.6 (1.1) | 173 | 5.6 (1.0) | 0.65 |
| Triglycerides (mmol/L) | 173 | 1.2 (0.9 - 1.8) | 178 | 1.1 (0.9 - 1.5) | 173 | 1.2 (0.9 - 1.5) | 0.041 |
| HDL cholesterol (mmol/L) | 173 | 1.5 (0.4) | 178 | 1.5 (0.4) | 173 | 1.5 (0.4) | 0.54 |
| LDL cholesterol (mmol/L) | 173 | 3.4 (0.9) | 178 | 3.4 (0.9) | 173 | 3.4 (1.0) | 0.71 |

**Table S3.** **Echocardiographic and ambulatory blood pressure measurements in NT-proANP tertiles in males and females.** n; number of, M; mean, SD; Standard deviation, IVS; intraventricular septum, PVW; posterior ventricular wall, LVM; left ventricular mass, LVMi; left ventricular mass index, FS; fractional shortening, LVid; left ventricular internal diameter, LAd; left atrial diameter, E/A; early to atrial filling velocity; SBP; systolic blood pressure, DBP; diastolic blood pressure, HR; heart rate. One-way ANOVA was used for tertile comparisons.

|  | Tertile 1 | | Tertile 2 | | Tertile 3 | |  |
| --- | --- | --- | --- | --- | --- | --- | --- |
| **Males** | n | M(SD) | n | M(SD) | n | M(SD) | *P* |
| IVS (mm) | 155 | 11.5 (2.0) | 156 | 11.5 (2.2) | 154 | 12.0 (2.3) | 0.042 |
| PVW (mm) | 155 | 10.6 (1.8) | 156 | 10.7 (1.7) | 154 | 11.2 (1.8) | 0.005 |
| LVM (g) | 155 | 283.6 (84.5) | 156 | 285.0 (80.4) | 154 | 309.8 (93.3) | 0.047 |
| LVMi (g/m²) | 155 | 141.2 (40.3) | 156 | 142.1 (36.5) | 154 | 155.5 (44.8) | 0.012 |
| FS (%) | 155 | 33.6 (5.2) | 156 | 34.5 (5.3) | 154 | 33.8 (6.8) | 0.34 |
| LVid | 155 | 53.6 (4.8) | 156 | 53.9 (5.5) | 154 | 54.3 (5.8) | 0.56 |
| LAd (mm) | 147 | 39.1 (4.8) | 151 | 40.7 (5.1) | 142 | 41.1 (5.5) | 0.003 |
| E/A ratio | 138 | 1.8 (0.6) | 151 | 1.8 (0.5) | 136 | 1.7 (0.6) | 0.063 |
| 24 h SBP (mmHg) | 146 | 133 (13) | 158 | 130 (12) | 142 | 130 (13) | 0.263 |
| 24 h DBP (mmHg) | 146 | 85 (8) | 158 | 83 (8) | 142 | 82 (8) | 0.023 |
| 24 h HR (bpm) | 146 | 72 (11) | 158 | 68 (10) | 142 | 67 (10) | <0.001 |
| **Females** | n | M(SD) | n | M(SD) | n | M(SD) | *P* |
| IVS (mm) | 157 | 10.1 (1.8) | 167 | 9.7 (1.6) | 159 | 10.0 (2.1) | 0.084 |
| PVW (mm) | 156 | 9.6 (1.4) | 166 | 9.3 (1.6) | 157 | 9.6 (1.8) | 0.12 |
| LVM (g) | 156 | 206.2 (55.2) | 166 | 196.3 (53.7) | 157 | 207.7 (63.8) | 0.15 |
| LVMi (g/m²) | 156 | 116.8 (28.0) | 166 | 112.6 (26.9) | 157 | 120.7 (34.3) | 0.051 |
| FS (%) | 156 | 35.8 (5.6) | 166 | 36.1 (5.8) | 157 | 35.8 (5.9) | 0.87 |
| LVid | 156 | 49.3 (4.0) | 166 | 49.5 (4.1) | 157 | 49.5 (4.8) | 0.86 |
| LAd (mm) | 146 | 37.7 (4.8) | 158 | 37.0 (5.1) | 151 | 38.8 (5.1) | 0.007 |
| E/A ratio | 145 | 1.6 (0.5) | 153 | 1.7 (0.6) | 149 | 1.7 (0.6) | 0.099 |
| 24 h SBP (mmHg) | 145 | 130 (14) | 159 | 126 (13) | 153 | 130 (15) | 0.039 |
| 24 h DBP (mmHg) | 145 | 81 (8) | 159 | 78 (8) | 153 | 79 (9) | 0.032 |
| 24 h HR (bpm) | 145 | 74 (9) | 159 | 71 (9) | 153 | 69 (9) | <0.001 |

**Table S4. Characteristics of the study population according to liver adiposity status.** n; number of, M; Mean, SD; Standard deviation, Mdn; Median, IQR; interquartile range, NT-proANP; N-terminal pro-atrial natriuretic peptide, BMI; body mass index, yrs; years, wk; week, SBP; systolic blood pressure, DBP; diastolic blood pressure, HR; heart rate , eGFR; estimated glomerular filtration rate, HOMA-IR; homeostatic model assessment for insulin resistance, HDL; high-density lipoprotein, LDL; low-density lipoprotein, HTN; hypertension, CAD; coronary artery disease, AMI; acute myocardial infarction, TIA; transient ischemic attack, CKD; chronic kidney disease, DM; diabetes mellitus, ASA; acetyl salicylic acid. For tertile comparisons, one-way ANOVA was used for normally distributed, Kruskal-Wallis’s test for non-normally distributed variables and Pearson’s Chi-square for categorical variables.

|  | No liver fat accumulation | | Liver fat accumulation | |  | |  |
| --- | --- | --- | --- | --- | --- | --- | --- |
|  | n | M(SD)/Mdn(IQR) | n | M(SD)/Mdn(IQR) | | *P* | |
| NT-proANP* (pmol/L) | 747 | 253 (182 - 348) | 280 | 237 (168 - 323) | | 0.053 | |
| Males, n (%) | 747 | 336 (45.0) | 280 | 172 (61.4) | | <0.001 | |
| Age (yrs) | 747 | 51.0 (6.0) | 280 | 51.8 (5.8) | | 0.065 | |
| Smoking* (pack yrs) | 747 | 0 (0 - 14) | 280 | 6 (0 - 22) | | <0.001 | |
| Alcohol consumption* (g/wk) | 747 | 20 (2 - 72) | 280 | 48 (3 - 131) | | <0.001 | |
| Diagnosis of HTN, n (%) | 747 | 331 (44.3) | 280 | 199 (71.1) | | <0.001 | |
| Diagnosis of CAD, n (%) | 747 | 74 (9.9) | 280 | 44 (15.7) | | 0.009 | |
| Diagnosis of CKD, n (%) | 747 | 32 (4.3) | 280 | 8 (2.9) | | 0.29 | |
| Diagnosis of DM, n (%) | 747 | 29 (3.9) | 280 | 76 (27.1) | | <0.001 | |
| Prior AMI, n (%) | 747 | 13 (1.7) | 280 | 10 (3.6) | | 0.077 | |
| Prior Stroke or TIA, n (%) | 747 | 28 (3.7) | 280 | 10 (3.6) | | 0.89 | |
| Beta blocker users, n (%) | 747 | 168 (22.5) | 280 | 113 (40.4) | | <0.001 | |
| BP medication users, n (%) | 747 | 334 (44.7) | 280 | 198 (70.7) | | <0.001 | |
| Lipid medication users, n (%) | 747 | 19 (2.5) | 280 | 11 (3.9) | | 0.24 | |
| ASA users, n (%) | 747 | 39 (5.2) | 280 | 16 (5.7) | | 0.75 | |
| BMI (kg/m2) | 747 | 26.5 (3.9) | 280 | 30.9 (5.0) | | <0.001 | |
| 24 h SBP (mmHg) | 653 | 128 (13) | 237 | 134 (14) | | <0.001 | |
| 24 h DBP (mmHg) | 653 | 80 (8) | 237 | 82 (8) | | 0.002 | |
| 24 h HR (bpm) | 653 | 70 (9) | 237 | 71 (11) | | 0.30 | |
| Leucocytes (E9/L) | 747 | 5.4 (4.6 - 6.4) | 280 | 6.1 (5.1 - 7.3) | | <0.001 | |
| Hemoglobin (g/L) | 747 | 140.2 (12.8) | 280 | 147.8 (12.0) | | <0.001 | |
| eGFR (mL/min/1.73m^3^) | 745 | 84.2 (15.3) | 280 | 84.7 (15.2) | | 0.68 | |
| Fasting glucose (mmol/l) | 747 | 4.3 (4.0 - 4.6) | 280 | 4.8 (4.4 - 5.7) | | <0.001 | |
| Fasting insulin (mmol/l) | 747 | 9.1 (6.8 - 12.8) | 280 | 18.2 (12.6 - 25.8) | | <0.001 | |
| HOMA-IR | 747 | 1.8 (1.3 - 2.6) | 280 | 4.1 (2.7 - 6.5) | | <0.001 | |
| Total cholesterol (mmol/L) | 747 | 5.7 ( 1.0) | 280 | 5.8 (1.1) | | 0.037 | |
| Triglycerides (mmol/L) | 747 | 1.2 (0.9 - 1.6) | 280 | 1.8 (1.3 - 2.4) | | <0.001 | |
| HDL cholesterol (mmol/L) | 747 | 1.4 (0.4) | 280 | 1.2 (0.3) | | <0.001 | |
| LDL cholesterol (mmol/L) | 747 | 3.5 (0.9) | 280 | 3.6 (1.0) | | 0.13 | |

**Table S5. Characteristics of the study subjects without CVDs, CKD, DM or related medications according to liver adiposity status.** n; number of, M; Mean, SD; Standard deviation, Mdn; Median, IQR; interquartile range, NT-proANP; N-terminal pro-atrial natriuretic peptide, BMI; body mass index, yrs; years, wk; week, SBP; systolic blood pressure, DBP; diastolic blood pressure, HR; heart rate , eGFR; estimated glomerular filtration rate, HOMA-IR; homeostatic model assessment for insulin resistance, HDL; high-density lipoprotein, LDL; low-density lipoprotein, For tertile comparisons, one-way ANOVA was used for normally distributed, Kruskal-Wallis’s test for non-normally distributed variables and Pearson’s Chi-square for categorical variables.

|  | No liver fat accumulation | | Liver fat accumulation | |  | |  |
| --- | --- | --- | --- | --- | --- | --- | --- |
|  | n | M(SD)/Mdn(IQR) | n | M(SD)/Mdn(IQR) | | *P* | |
| NT-proANP (pmol/L) | 350 | 233 (177 - 317) | 53 | 185 (154 - 252) | | 0.001 | |
| Males, n (%) | 350 | 161 (46.0) | 53 | 35 (66.0) | | 0.007 | |
| Age (yrs) | 350 | 50.3 (5.9) | 53 | 51.7 (5.9) | | 0.12 | |
| Smoking (pack yrs) | 350 | 0 (0 - 14) | 53 | 10 (0 - 29) | | 0.001 | |
| Alcohol consumption (g/wk) | 350 | 18 (2 - 72) | 53 | 48 (12 - 144) | | 0.002 | |
| BMI (kg/m2) | 350 | 25.5 (3.4) | 53 | 29.9 (4.7) | | <0.001 | |
| 24 h SBP (mmHg) | 305 | 125 (12) | 44 | 132 (16) | | <0.001 | |
| 24 h DBP (mmHg) | 305 | 78 (8) | 44 | 82 (8) | | 0.003 | |
| 24 h HR (bpm) | 305 | 71 (9) | 44 | 72 (9) | | 0.63 | |
| Leucocytes (E9/L) | 350 | 5.3 (4.4 - 6.3) | 53 | 5.8 (5.0 - 7.1) | | 0.007 | |
| Hemoglobin (g/L) | 350 | 139.3 (12.8) | 53 | 148.6 (12.1) | | 0.001 | |
| eGFR (mL/min/1.73m^3^) | 348 | 87.0 (14.0) | 53 | 87.9 (12.5) | | 0.66 | |
| Fasting glucose (mmol/l) | 350 | 4.3 (4.0 - 4.5) | 53 | 4.4 (4.2 - 5.0) | | <0.001 | |
| Fasting insulin (mmol/l) | 350 | 8.3 (6.3 - 11.3) | 53 | 17.9 (10.5 - 24.6) | | <0.001 | |
| HOMA-IR | 350 | 1.6 (1.2 - 2.3) | 53 | 3.4 (2.1 - 4.9) | | <0.001 | |
| Total cholesterol (mmol/L) | 350 | 5.5 (1.0) | 53 | 5.9 (1.0) | | 0.013 | |
| Triglycerides (mmol/L) | 350 | 1.1 (0.9 - 1.4) | 53 | 1.7 (1.2 - 2.5) | | <0.001 | |
| HDL cholesterol (mmol/L) | 350 | 1.4 (0.4) | 53 | 1.2 (0.4) | | <0.001 | |
| LDL cholesterol (mmol/L) | 350 | 3.4 (0.9) | 53 | 3.7 (0.8) | | 0.049 | |

**Table S6. Characteristics of the study subjects with CVDs, CKD, DM or related medications according to liver adiposity status.** n; number of, M; Mean, SD; Standard deviation, Mdn; Median, IQR; interquartile range, NT-proANP; N-terminal pro-atrial natriuretic peptide, BMI; body mass index, yrs; years, wk; week, SBP; systolic blood pressure, DBP; diastolic blood pressure, HR; heart rate , eGFR; estimated glomerular filtration rate, HOMA-IR; homeostatic model assessment for insulin resistance, HDL; high-density lipoprotein, LDL; low-density lipoprotein, For tertile comparisons, one-way ANOVA was used for normally distributed, Kruskal-Wallis’s test for non-normally distributed variables and Pearson’s Chi-square for categorical variables.

|  | No liver fat accumulation | | Liver fat accumulation | |  |
| --- | --- | --- | --- | --- | --- |
|  | n | M(SD)/Mdn(IQR) | n | M(SD)/Mdn(IQR) | *P* |
| NT-proANP (pmol/L) | 397 | 272 (184 - 365) | 227 | 247 (172 - 347) | 0.091 |
| Males, n (%) | 397 | 175 (44.1) | 227 | 137 (60.4) | <0.001 |
| Age (yrs) | 397 | 51.7 (6.1) | 227 | 51.8 (5.8) | 0.75 |
| Smoking (pack yrs) | 397 | 0 (0 - 14) | 227 | 5 (0 - 22) | 0.01 |
| Alcohol consumption* (g/wk) | 397 | 24 (1 - 70) | 227 | 48 (3 - 123) | <0.001 |
| Diagnosis of HTN, n (%) | 397 | 331 (83.4) | 227 | 199 (87.7) | 0.15 |
| Diagnosis of CAD, n (%) | 397 | 74 (18.6) | 227 | 44 (19.4) | 0.82 |
| Diagnosis of CKD, n (%) | 397 | 32 (8.1) | 227 | 8 (3.5) | 0.026 |
| Diagnosis of DM, n (%) | 397 | 29 (7.3) | 227 | 76 (33.5) | <0.001 |
| Prior AMI, n (%) | 397 | 13 (3.3) | 227 | 10 (4.4) | 0.47 |
| Prior Stroke or TIA, n (%) | 397 | 28 (7.1) | 227 | 10 (4.4) | 0.18 |
| BP medication users, n (%) | 397 | 334 (84.1) | 227 | 198 (87.2) | 0.29 |
| Lipid medication users, n (%) | 397 | 19 (4.8) | 227 | 11 (4.8) | 0.97 |
| BMI (kg/m2) | 397 | 27.4 (4.1) | 227 | 31.1 (5.0) | <0.001 |
| 24 h Systolic ABP (mmHg) | 348 | 131 (13) | 193 | 134 (14) | 0.01 |
| 24 h Diastolic ABP (mmHg) | 348 | 82 (9) | 193 | 82 (8) | 0.71 |
| 24 h HR (bpm) | 348 | 69 (10) | 193 | 71 (11) | 0.10 |
| Leucocytes (E9/L) | 397 | 5.6 (4.8 - 6.6) | 227 | 6.1 (5.2 - 7.4) | <0.001 |
| Hemoglobin (g/L) | 397 | 141.0 (12.7) | 227 | 147.6 (12.0) | <0.001 |
| eGFR (mL/min/1.73m^3^) | 397 | 81.7 (15.9) | 227 | 83.9 (15.7) | 0.10 |
| Fasting glucose (mmol/l) | 397 | 4.4 (4.1 - 4.7) | 227 | 4.9 (4.5 - 5.8) | <0.001 |
| Fasting insulin (mmol/l) | 397 | 10.0 (7.3 - 14.4) | 227 | 18.3 (12.6 - 26.2) | <0.001 |
| HOMA-IR | 397 | 1.9 (1.4 - 2.9) | 227 | 4.2 (2.7 - 7.0) | <0.001 |
| Total cholesterol (mmol/L) | 397 | 5.8 (1.1) | 227 | 5.8 (1.1) | 0.71 |
| Triglycerides (mmol/L) | 397 | 1.3 (1.0 - 1.8) | 227 | 1.8 (1.4 - 2.4) | <0.001 |
| HDL cholesterol (mmol/L) | 397 | 1.4 (0.4) | 227 | 1.2 (0.3) | <0.001 |
| LDL cholesterol (mmol/L) | 397 | 3.6 (1.0) | 227 | 3.6 (1.0) | 0.86 |

**Table S7. Logistic regression model for liver fat accumulation at baseline in the study population and subjects without and with CVDs, CKD, DM or related medications.** Odds ratios (OR) with 95% Confidence Intervals (CI) for all variables in the model. NT-proANP; N-terminal pro-atrial natriuretic peptide, g/wk; grams per week, BMI; body mass index, HOMA-IR; homeostatic model assessment for insulin resistance, Hb, hemoglobin.

| **All subjects** | OR (95% CI) |
| --- | --- |
| NT-proANP tertiles | 0.83 [0.67; 1.03] |
| Alcohol consumption (g/wk) | 1.002 [1.000; 1.004] |
| HOMA-IR | 1.28 [1.16; 1.40] |
| Triglycerides (mmol/L) | 1.21 [1.01; 1.46] |
| BMI (kg/m2) | 1.13 [1.08; 1.18] |
| Antihypertensive use | 1.68 [1.17; 2.41] |
| Diabetes | 3.40 [1.94; 5.94] |
| Hb (g/L) | 1.03 [1.01; 1.04] |
| **Without CVDs** | OR (95% CI) |
| NT-proANP tertiles | 0.84 [0.55; 1.28] |
| HOMA-IR | 1.42 [1.15; 1.75] |
| Hb (g/L) | 1.04 [1.02; 1.08] |
| BMI (kg/m2) | 1.23 [1.12; 1.34] |
| **With CVDs** | OR (95% CI) |
| NT-proANP tertiles | 0.81 (0.64 – 1.03) |
| Alcohol consumption (g/wk) | 1.002 (1.000 – 1.005) |
| Diabetes | 3.23 (1.85 – 5.67) |
| BMI (kg/m2) | 1.11 (1.06 – 1.17) |
| Hb (g/L) | 1.03 (1.01 – 1.04) |
| HOMA-IR | 1.27 (1.14 – 1.41) |

**Table S8. Number of heart failure and CVD events (until 2014), CVD mortality (until 2021) and total mortality (until 2021) in NT-proANP tertiles in males, females and subjects without and with CVDs, CKD, DM or related medications.** CVD; cardiovascular disease, n, number of subjects.

| **All subjects** | Tertile 1 | Tertile 2 | Tertile 3 | Total |
| --- | --- | --- | --- | --- |
| n | 345 | 353 | 346 | 1044 |
| Heart failure n (%) | 15 (4.3) | 13 (3.7) | 42 (12.1) | 70 (6.7) |
| CVD events n (%) | 74 (21.4) | 82 (23.2) | 99 (28.6) | 255 (24.4) |
| CVD mortality n (%) | 37 (10.7) | 37 (10.5) | 65 (18.8) | 139 (13.3) |
| Total mortality n (%) | 119 (34.5) | 131 (37.1) | 163 (47.1) | 413 (39.6) |
| **Males** | Tertile 1 | Tertile 2 | Tertile 3 | Total |
| n | 172 | 175 | 173 | 520 |
| Heart failure n (%) | 11 (6.4) | 7 (4.0) | 27 (15.6) | 45 (8.7) |
| CVD events n (%) | 49 (28.5) | 58 (33.1) | 65 (37.6) | 172 (33.1) |
| CVD mortality n (%) | 25 (14.5) | 29 (16.6) | 43 (24.9) | 97 (18.7) |
| Total mortality n (%) | 69 (40.1) | 83 (47.4) | 99 (57.2) | 251 (48.3) |
| **Females** | Tertile 1 | Tertile 2 | Tertile 3 | Total |
| n | 173 | 178 | 173 | 524 |
| Heart failure n (%) | 4 (2.3) | 6 (3.4) | 15 (8.7) | 25 (4.8) |
| CVD events n (%) | 25 (14.5) | 24 (13.5) | 35 (20.2) | 83 (15.8) |
| CVD mortality n (%) | 12 (6.9) | 8 (4.5) | 22 (12.7) | 42 (8.0) |
| Total mortality n (%) | 50 (28.9) | 48 (27.0) | 64 (37.0) | 162 (30.9) |
| **Without CVDs** | Tertile 1 | Tertile 2 | Tertile 3 | Total |
| n | 138 | 136 | 136 | 410 |
| Heart failure n (%) | 1 (0.7) | 2 (1.5) | 10 (7.4) | 13 (3.2) |
| CVD events n (%) | 23 (16.7) | 14 (10.3) | 27 (19.9) | 64 (15.6) |
| CVD mortality n (%) | 11 (8.0) | 8 (5.9) | 18 (13.2) | 37 (9.0) |
| Total mortality n (%) | 44 (31.9) | 45 (34.1) | 47 (34.5) | 136 (33.1) |
| **With CVDs** | Tertile 1 | Tertile 2 | Tertile 3 | Total |
| n | 213 | 210 | 211 | 634 |
| Heart failure n (%) | 14 (6.6) | 16 (7.6) | 27 (12.8) | 57 (9.0) |
| CVD events n (%) | 55 (25.8) | 68 (32.4) | 68 (32.2) | 191 (30.1) |
| CVD mortality n (%) | 28 (13.1) | 27 (12.9) | 47 (22.3) | 102 (16.1) |
| Total mortality n (%) | 78 (36.6) | 86 (41.0) | 113 (53.6) | 277 (43.7) |

**Table S9.** Hazard ratios (HR) for heart failure, CVD events, CVD mortality and total mortality according to NT-proANP tertiles in all subjects and subjects without and with CVDs, CKD, DM or related medications. CI; 95% confidence interval, CVD; cardiovascular disease, g/wk; grams per week, BMI; body mass index, eGFR; estimated glomerular filtration rate, HOMA-IR; homeostatic model assessment for insulin resistance, SBP; systolic blood pressure, ASA; acetyl salicylic acid.

|  | Heart failure | CVD events | CVD mortality | Total mortality |
| --- | --- | --- | --- | --- |
|  | HR (95 % CI) | HR (95 % CI) | HR (95 % CI) | HR (95 % CI) |
| **All subjects** |  |  |  |  |
| NT-proANP tertile 1 (Low) | 1 | 1 | 1 | 1 |
| NT-proANP tertile 2 | 0.63 (0.34 – 1.41) | 1.10 (0.84 – 1.63) | 1.12 (0.62 – 1.81) | 1.00 (0.82 – 1.30) |
| NT-proANP tertile 3 (High) | 2.21 (1.22 – 4.33) | 1.42 (0.92 – 1.92) | 1.90 (1.22 – 3.27) | 1.23 (0.91 – 1.63) |
| Sex (female) | 1.12 (0.63 – 2.12) | 0.62 (0.30 – 0.83) | 0.73 (0.40 – 1.06) | 0.82 (0.65 – 1.05) |
| Age (years) | 1.08 (1.02 – 1.14) | 1.07 (0.42 – 1.09) | 1.08 (1.04 – 1.12) | 1.13 (1.08 – 1.13) |
| Smoking (pack-years) | 1.00 (0.99 – 1.01) | 1.01 (1.00 – 1.02) | 1.02 (1.01 – 1.03) | 1.02 (1.01 – 1.03) |
| Alcohol consumption (g/wk) | 1.00 (1.00 – 1.00) | 1.00 (0.99 – 1.00) | 1.00 (1.00 – 1.00) | 1.00 (1.00 – 1.00) |
| BMI (kg/m^2^) | 1.06 (0.99 – 1.12) | 0.98 (0.94 – 1.01) | 1.02 (0.98 – 1.07) | 0.99 (0.97 – 1.02) |
| 24 h SBP (mmHg) | 1.00 (0.99 – 1.03) | 1.02 (1.00 – 1.03) | 1.02 (1.00 – 1.03) | 1.01 (1.00 – 1.02) |
| eGFR (mL /min/1.73m^3^) | 1.02 (1.00 – 1.04) | 1.00 (0.99 – 1.02) | 1.00 (0.99 – 1.02) | 1.00 (0.99 – 1.01) |
| HOMA-IR | 1.01 (0.95 – 1.07) | 1.04 (1.00 – 1.08) | 1.04 (1.02 – 1.10) | 1.06 (1.03 – 1.09) |
| Triglycerides (mmol/L) | 1.20 (0.96 – 1.63) | 1.37 (1.21 – 1.62) | 1.31 (1.09 – 1.63) | 1.10 (0.97 – 1.32) |
| LDL cholesterol (mmol/L) | 0.99 (0.76 – 1.30) | 1.22 (1.05 – 1.42) | 1.24 (1.01 – 1.55) | 1.03 (0.92 – 1.23) |
| Antihypertensive medication | 1.12 (0.64 – 2.09) | 1.43 (1.04 – 1.83) | 0.98 (0.61 – 1.54) | 0.97 (0.84 – 1.25) |
| ASA | 1.64 (0.73 – 3.72) | 1.04 (0.61 – 1.72) | 1.10 (0.53 – 2.12) | 1.18 (0.84 – 1.86) |
| **Without CVDs** |  |  |  |  |
| NT-proANP tertile 1 (Low) | 1 | 1 | 1 | 1 |
| NT-proANP tertile 2 | 1.90 (0.22 – 22.81) | 0.97 (0.53 – 2.11) | 1.92 (0.62 – 5.91) | 1.11 (0.71 – 1.82) |
| NT-proANP tertile 3 (High) | 8.05 (0.89 – 75.42) | 1.91 (0.94 – 3.93) | 5.12 (1.62 – 16.43) | 1.12 (0.72 – 1.93) |
| Sex (female) | 2.15 (0.53 – 8.94) | 0.63 (0.33 – 1.22) | 0.61 (0.33 – 1.54) | 0.62 (0.41 – 1.13) |
| Age (years) | 1.21 (1.03 – 1.32) | 1.05 (0.99 – 1.10) | 1.08 (1.00 – 1.16) | 1.14 (1.06 – 1.14) |
| Smoking (pack-years) | 1.03 (1.00 – 1.06) | 1.02 (1.00 – 1.04) | 1.04 (1.01 – 1.06) | 1.04 (1.02 – 1.05) |
| Alcohol consumption (g/wk) | 1.00 (0.99 – 1.01) | 0.99 (0.99 – 1.01) | 0.99 (0.99 – 1.01) | 0.99 (0.99 – 1.01) |
| BMI (kg/m^2^) | 1.03 (0.94 – 1.22) | 1.02 (0.94 – 1.15) | 1.02 (0.92 – 1.16) | 1.00 (0.95 - 1.07) |
| 24 h SBP (mmHg) | 1.03 (0.99 – 1.07) | 1.04 (1.02 – 1.06) | 1.04 (1.01 – 1.07) | 1.02 (1.01 – 1.04) |
| eGFR (mL /min/1.73m^3^) | 1.00 (0.96 – 1.04) | 1.01 (0.99 – 1.03) | 0.99 (0.97 – 1.02) | 1.01 (0.99 – 1.02) |
| HOMA-IR | 0.95 (0.64 – 1.52) | 1.01 (0.86 – 1.20) | 1.2 (1.03 – 1.40) | 1.07 (0.97 – 1.18) |
| Triglycerides (mmol/L) | 1.27 (0.52 – 3.34) | 1.57 (1.09 – 2.26) | 1.32 (0.8 1– 2.21) | 1.08 (0.81 – 1.43) |
| LDL cholesterol (mmol/L) | 0.75 (0.33 – 1.63) | 1.02 (0.74 – 1.42) | 1.42 (0.93 – 2.23) | 1.02 (0.84 – 1.32) |
| **With CVDs** |  |  |  |  |
| NT-proANP tertile 1 (Low) | 1 | 1 | 1 | 1 |
| NT-proANP tertile 2 | 0.87 (0.41 - 1.85) | 1.24 (0.88 - 1.77) | 1.06 (0.63 - 1.77) | 1.08 (0.82 - 1.41) |
| NT-proANP tertile 3 (High) | 2.12 (1.09 - 4.13) | 1.34 (0.94 - 1.92) | 2.15 (1.34 - 3.46) | 1.27 (0.97 - 1.68) |
| Sex (female) | 1.09 (0.56 - 2.10) | 0.56 (0.40 - 0.79) | 0.66 (0.41 - 1.06) | 0.82 (0.63 - 1.06) |
| Age (years) | 1.08 (1.03 - 1.14) | 1.07 (1.04 - 1.09) | 1.08 (1.04 - 1.13) | 1.11 (1.08 - 1.13) |
| Smoking (pack-years) | 1.01 (0.99 - 1.02) | 1.01 (1.00 - 1.02) | 1.02 (1.01 - 1.03) | 1.02 (1.01 - 1.03) |
| Alcohol consumption (g/wk) | 1.00 (1.00 - 1.00) | 1.00 (1.00 - 1.00) | 1.00 (1.00 - 1.00) | 1.00 (1.00 - 1.00) |
| BMI (kg/m^2^) | 1.05 (0.99 - 1.12) | 0.98 (0.94 - 1.01) | 1.03 (0.98 - 1.07) | 1.00 (0.97 - 1.02) |
| 24 h SBP (mmHg) | 1.01 (0.99 - 1.03) | 1.02 (1.01 - 1.03) | 1.02 (1.01 - 1.03) | 1.01 (1.00 - 1.02) |
| eGFR (mL /min/1.73m^3^) | 1.02 (1.00 - 1.04) | 1.01 (1.00 - 1.02) | 1.00 (0.99 - 1.02) | 1.01 (1.00 - 1.01) |
| HOMA-IR | 1.01 (0.95 - 1.07) | 1.04 (1.00 - 1.08) | 1.06 (1.01 - 1.10) | 1.06 (1.03 - 1.09) |
| Triglycerides (mmol/L) | 1.23 (0.97 - 1.56) | 1.37 (1.19 - 1.57) | 1.31 (1.09 - 1.57) | 1.10 (0.97 - 1.25) |
| LDL cholesterol (mmol/L) | 1.00 (0.77 - 1.31) | 1.22 (1.05 - 1.42) | 1.24 (1.01 - 1.52) | 1.03 (0.92 - 1.16) |
| Antihypertensive medication | 1.23 (0.69 - 2.18) | 1.38 (1.03 - 1.87) | 1.00 (0.67 - 1.50) | 0.98 (0.78 - 1.23) |
| ASA | 1.68 (0.73 - 3.85) | 0.98 (0.57 - 1.67) | 1.08 (0.54 - 2.17) | 1.19 (0.80 - 1.78) |

**Table S10.** **Characteristics of the study subjects without CVDs, CKD, DM or related medications according to CVD mortality during follow-up.** n; number of, M; Mean, SD; Standard deviation, Mdn; Median, IQR; interquartile range, NT-proANP; N-terminal pro-atrial natriuretic peptide, BMI; body mass index, yrs; years, g/wk; grams per week; SBP; systolic blood pressure, DBP; diastolic blood pressure, HR; heart rate, hsCRP, eGFR; estimated glomerular filtration rate, HOMA-IR; homeostatic model assessment for insulin resistance, HDL; high-density lipoprotein, LDL; low-density lipoprotein. For tertile comparisons, one-way ANOVA was used for normally distributed, Kruskal-Wallis’s test for non-normally distributed variables and Pearson’s Chi-square for categorical variables.

|  | No CVD death | | CVD death | | *P* |
| --- | --- | --- | --- | --- | --- |
| **Healthy subjects** | N | M(SD)/Mn(IQR) | N | M(SD)/Mn(IQR) |  |
| NT-proANP (pmol/L) | 373 | 225 (175 - 305) | 37 | 258 (170 -373) | 0.20 |
| Males, n (%) | 373 | 177 (47.5) | 37 | 25 (67.6) | 0.024 |
| Age (yrs) | 373 | 50.1 (5.8) | 37 | 54.2 (5.9) | < 0.001 |
| Smoking (pack yrs) | 373 | 1 (0 -14.0) | 37 | 13 (0 - 30.0) | 0.015 |
| Alcohol consumption (g/wk) | 373 | 23 (3.0 - 83.5) | 37 | 36 (7.5 - 79.5) | 0.40 |
| BMI (kg/m2) | 373 | 26.0 (3.8) | 37 | 27.2 (4.2) | 0.068 |
| eGFR (mL/min/1.73m^3^) | 371 | 84.9 (16.6) | 37 | 82.0 (11.4) | 0.29 |
| Fasting glucose (mmol/l) | 373 | 4.3 (0.4) | 37 | 4.4 (0.5) | 0.15 |
| Fasting insulin (mmol/l) | 373 | 8.7 (6.7 - 12.2) | 37 | 9.8 (6.5 - 19.0) | 0.22 |
| Fatty liver at baseline n (%) | 373 | 40 (10.7) | 37 | 13 (36.1) | <0.001 |
| Total cholesterol (mmol/L) | 373 | 5.6 (1.0) | 37 | 5.8 (0.9) | 0.17 |
| Triglycerides (mmol/L) | 373 | 1.1 (0.9 - 1.5) | 37 | 1.5 (1.1 - 1.8) | 0.001 |
| HDL cholesterol (mmol/L) | 373 | 1.4 (0.4) | 37 | 1.3 (0.4) | 0.020 |
| LDL cholesterol (mmol/L) | 373 | 3.4 (0.9) | 37 | 3.7 (0.8) | 0.059 |
| 24 h SBP (mmHg) | 322 | 125 (12) | 33 | 133 (14) | < 0.001 |
| 24 h DBP (mmHg) | 322 | 79 (8) | 33 | 81 (10) | 0.13 |
| 24 h HR (bpm) | 322 | 72 (9) | 33 | 70 (10) | 0.47 |

**Figure S1. Flow chart representing the study population and analyses done.** OPERA; Oulu Project Elucidating Risk of Atherosclerosis, n; number of, NT-proANP; N-terminal proatrial natriuretic peptide, BP; blood pressure, T; tertile, CVD; cardiovascular disease, DM; diabetes, CKD; chronic kidney disease.

**Figure S2.** **Probabilities of event-free follow-up and survival probabilities according to NT-proANP tertiles, in all subjects and subjects with and without CVDs, CKD, DM, or related medications.** Kaplan-Meier graphs represent survival probabilities for (A, B, C) Heart failure, (D, E, F) CVD events, (G, H, I) CVD mortality, and (J, K, L) Total mortality in each NT-proANP tertile. CVD, cardiovascular disease. (A, D, G, J) represent all subjects (B, E, H, K) study subjects without CVDs, CKD, DM, or related medications , and (C, F, I, L) subjects with CVDs, CKD, DM, or related medications. NT-proANP tertile 1 has the lowest and NT-proANP tertile 3 the highest NT-proANP levels.
